# MicroRNA-141 suppresses prostate cancer stem cells and metastasis by targeting a cohort of pro-metastasis genes

**DOI:** 10.1038/ncomms14270

**Published:** 2017-01-23

**Authors:** Can Liu, Ruifang Liu, Dingxiao Zhang, Qu Deng, Bigang Liu, Hsueh-Ping Chao, Kiera Rycaj, Yoko Takata, Kevin Lin, Yue Lu, Yi Zhong, John Krolewski, Jianjun Shen, Dean G. Tang

**Affiliations:** 1 Department of Epigenetics and Molecular Carcinogenesis, University of Texas MD Anderson Cancer Center, Science Park, Texas 78957, USA; 2 Cancer Stem Cell Institute, Research Center for Translational Medicine, East Hospital, Tongji University School of Medicine, Shanghai 200120, China; 3 Department of Pharmacology and Therapeutics, Roswell Park Cancer Institute, Carlton and Elm Streets, Buffalo, New York 14263, USA; 4 Department of Epigenetics and Molecular Carcinogenesis, Program in Molecular Carcinogenesis, University of Texas Graduate School of Biomedical Sciences (GSBS), Houston, Texas 77030, USA; 5 Department of Cancer Genetics, Roswell Park Cancer Institute, Buffalo, New York 14263, USA

## Abstract

MicroRNAs play important roles in regulating tumour development, progression and metastasis. Here we show that one of the miR-200 family members, miR-141, is under-expressed in several prostate cancer (PCa) stem/progenitor cell populations in both xenograft and primary patient tumours. Enforced expression of miR-141 in CD44^+^ and bulk PCa cells inhibits cancer stem cell properties including holoclone and sphere formation, as well as invasion, and suppresses tumour regeneration and metastasis. Moreover, miR-141 expression enforces a strong epithelial phenotype with a partial loss of mesenchymal phenotype. Whole-genome RNA sequencing uncovers novel miR-141-regulated molecular targets in PCa cells including the Rho GTPase family members (for example, CDC42, CDC42EP3, RAC1 and ARPC5) and stem cell molecules CD44 and EZH2, all of which are validated as direct and functionally relevant targets of miR-141. Our results suggest that miR-141 employs multiple mechanisms to obstruct tumour growth and metastasis.

## Abstract (web summary)

MicroRNAs have important roles in regulating tumor development, progression and metastasis. Here, the authors demonstrate the tumor-suppressive functions of miRNA141 in prostate cancer stem cells mediated by directly targeting CD44, Rho GTPase protein family members, and EZH2.

Human cancers are heterogeneous containing cancer stem cells (CSCs) that possess high capacities for tumour propagation and metastasis^1^,^2^,^3^. Metastasis causes >90% of cancer-related deaths but our understanding of the molecular mechanisms that regulate metastasis remains limited. The invasion–metastasis cascade is a multistep cellular process that involves dissemination of cancer cells through the surrounding extracellular matrix, survival in the circulation and initial seeding followed by subsequent expansion (colonization) in the foreign microenvironment. Recent evidence suggests that microRNAs (miRNAs), small (∼20–22 nt) non-coding RNAs that modulate multiple biological processes, play important roles in regulating CSCs, tumour development and metastasis^4^,^5^,^6^,^7^. Specific miRNAs, highlighted by miR-34a, let-7, miR-10b, miR-93 and miR-200 family^8^,^9^,^10^,^11^,^12^,^13^,^14^,^15^,^16^,^17^ may function as either promoters or suppressors of metastasis via a variety of mechanisms.

In human prostate cancer (PCa), several CSC populations have been reported using cell surface markers (for example, CD44, CD133, integrin α2β1, ABCG2 and so on), functional assays including side population and Aldefluor, and reporter-based lineage tracing strategies^18^,^19^,^20^,^21^,^22^,^23^,^24^,^25^,^26^. These prostate CSC (PCSC) populations have been shown to possess high clonal, clonogenic, tumour-propagating, invasive and metastatic activities, and to be refractory to castration, Docetaxel, and many other therapeutics. Nevertheless, how PCSCs are molecularly regulated, for example, by miRNAs, remains poorly understood. In a previous miRNA library screening for PCSC-regulating miRNAs, we discovered that miR-34a and let-7, both being potent tumour suppressors, are prominently under-expressed in several PCSC populations and negatively regulate PCSC activity, tumour growth and metastasis^13^,^14^. In the same screening, we also identified miR-141, one of the miR-200 family members, to be significantly reduced in the CD44^+^ PCSC cells. However, there is as yet no systematic investigation on the functional role of miR-141 in regulating PCSCs, especially in the context of PCa progression and metastasis.

The miR-200 family, which encompasses miR-200a, b and c, miR-429 and miR-141, is among the first to be reported as important negative regulators of epithelial to mesenchymal transition (EMT)^8^,^9^,^10^, an essential developmental process implicated in cancer metastasis^27^,^28^. Although the prevailing view is that under-expression of miR-200s promotes EMT and metastasis, there are also reports of upregulated expression and potential metastasis-promoting effects of miR-200 members in different types or subtypes of cancer^11^,^29^. In addition, serum levels of miR-141 and other miR-200 family members have been positively associated with the different clinical outcomes of prostate, ovarian, colon and breast cancers^30^,^31^,^32^. These seemingly conflicting reports further prompted us to investigate the expression and function of miR-141 in PCa and PCSCs.

Herein, we report that miR-141 is under-expressed in CD44^+^ PCSCs from both xenograft and patient tumours, and miR-141 exhibits tumour and metastasis-suppressing effects in PCa. Whole-genome RNA sequencing (RNA-Seq) analysis identified multiple pro-metastasis genes including *CD44*, *Rho GTPase* and *EZH2* as direct and functionally relevant targets of miR-141.

## Results

### miR-141 is under-expressed in CD44^+^ PCa cells in patient tumours

**Figure 1:**
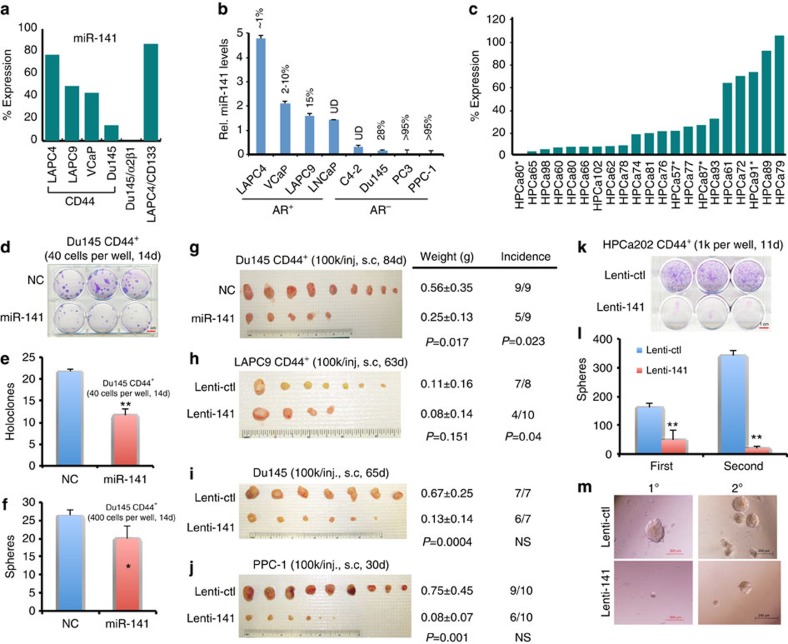
Underexpression of miR-141 in CD44^+^ PCa cells and its PCa-suppressive functions. (**a**) Relative expression levels of miR-141 in CSC populations (compared with the corresponding marker-negative cells) in xenografts including CD44^+^ populations from LAPC4, LAPC9, VCaP and DU145, α2β1^+^ from DU145 and CD133^+^ from LAPC4. (**b**) Relative expression levels of miR-141 in four AR^+^ and four AR^−^ PCa cell types. Average % of CD44^+^ cells (as determined by flow analysis; *n*=3–5) is indicated for each cell type (UD, undetectable). (**c**) Relative expression levels of miR-141 in CD44^+^ cells (as % of the corresponding CD44^−^ cells) from 21 primary HPCa samples. Samples marked by * indicate early generation PDX tumours. (**d**–**f**) miR-141 inhibits clonal and clonogenic properties of CD44^+^ DU145 cells as shown in clonal (**d**,**e**) and sphere (**f**) assays. (**g**–**j**) miR-141 inhibits prostate tumour regeneration and growth. Shown are tumour images, cell numbers injected (100,000 cells per injection for all models), site of injection (s.c, subcutaneously) and days (**d**) when tumours were collected. Tumour weight and incidences with corresponding *P*-values are also indicated. NS, not significant. (**k**–**m**) miR-141 inhibits clonal and clonogenic properties of primary CD44^+^ HPCa cells. Purified CD44^+^ HPCa202 cells were infected with Lenti-ctl or Lenti-141 and plated for clonal assay (**k**) or (serial) sphere assays in Matrigel (**l**,**m**). Quantification of the colonies from first (1°) and second (2°) generations was plotted (**l**) and representative images shown below (**m**). Values represent mean±s.d. of triplicates from three independent experiments. *P<0.05 and **P<0.01. Scale bar, 1 cm (**d**,**k**). *P* values were calculated using upaired student's *t*-test for panels **e** and **f**, tumor weight comparisons in panels **g**–**j**, and sphere numbers for panel l. *P* values for tumor incidences in panels **g**–**j** were calculated using Chi-square test. NS, not significant.

Systematic studies from our lab have established that the CD44^+^ PCa cell population is enriched in clonogenic and tumourigenic cells that fulfill the CSC definition^13^,^20^,^21^,^23^,^25^. In a previous miRNA expression profiling of a library of ∼310 sequence-validated human miRNAs^13^,^14^, we observed that miR-141 was significantly under-expressed in CD44^+^ and several other PCa stem/progenitor cell populations. To further explore this observation, we purified CD44^+^ PCa cells from LAPC9, LAPC4 and DU145 xenografts, VCaP cultures and, for comparisons, CD133^+^ cells^19^ from LAPC4 xenografts and integrin α2β1^+^ cells^21^,^25^ from DU145 xenografts, and performed quantitative reverse transcription–PCR (qRT–PCR) analysis (Supplementary Table 1) of mature miR-141 levels relative to the corresponding marker-negative populations. We found that miR-141 was commonly under-expressed in these PCa stem/progenitor populations, including all CD44^+^ subpopulations (Fig. 1a). Furthermore, correlation analysis in eight PCa xenograft/culture-derived cell types revealed that the miR-141 messenger RNA levels overall inversely correlated with the abundance of CD44^+^ cells (Fig. 1b). Importantly, the miR-141 levels were much lower in the CD44^+^ cells freshly purified from 21 primary human PCa (HPCa) patient samples (Supplementary Table 2) compared with the corresponding CD44^−^ HPCa cells (Fig. 1c). We further adopted a qRT–PCR approach to estimate the copy numbers of miR-141 in PCa cells and subpopulations (Supplementary Fig. 1 and see Methods). We observed that on normalizing to the standard curve (Supplementary Fig. 1a,b), the endogenous miR-141 levels in five PCa cell types varied widely (Supplementary Fig. 1c). Significantly, miR-141 levels in bulk HPCa cells from 20 patient tumour samples also varied widely ranging from barely detectable to ∼100,000 copies per cell (Supplementary Fig. 1d). Notably, in the majority of the 20 samples analysed, miR-141 levels were lower in CD44^+^ cells than in the corresponding CD44^−^ cells (Supplementary Fig. 1d,e).

### miR-141 inhibits PCSC properties and tumour regeneration

As the CD44^+^ PCa cells are prominently devoid of miR-141, we first restored its expression in CD44^+^ PCa cells and performed CSC assays including stringent clonal (holoclone) and single cell-derived sphere-formation assays^13^,^14^,^23^,^25^,^33^. Freshly purified CD44^+^ DU145 cells were transfected with the miR-141 mimicking (miR-141) or control (NC) oligos for 48 h and then used in clonal and sphere assays. Transfection of 30 nM miR-141 mimics for 48 h resulted in significantly increased, that is, ∼128-fold in PPC-1 cells to >67,000-fold in PC3 cells, miR-141 levels compared with the same cells transfected with NC oligos (Supplementary Fig. 1f). Direct measurement of miR-141 copy numbers in DU145 cells indicated that cultured DU145 cells expressed a wide range of endogenous miR-141 levels from barely detectable to ∼6,000 copies per cell (*n*=11 independent measurements; Supplementary Fig. 1c). Transfection of miR-141 oligos at 3–60 nM in DU145 cells resulted in ∼2–30-fold increases in miR-141 copy numbers compared with untransfected cells (Supplementary Fig. 1g). Under these experimental conditions, we observed that 30 nM miR-141 mimics inhibited both clonogenic (Fig. 1d,e) and sphere-forming (Fig. 1f) capacities of CD44^+^ DU145 cells. Importantly, miR-141 oligo transfection inhibited both tumour incidence and weight *in vivo* in CD44^+^ DU145 cells (Fig. 1g) and PPC-1 tumour regeneration (Supplementary Fig. 2a).

To validate the biological effects of miR-141 mimics, we constructed two lentiviral vectors to overexpress miR-141 (Supplementary Fig. 1h,i). Infection of three PCa cell types with the two lenti-vectors at a multiplicity of infection (MOI) of 20 for 48 h resulted in 14- to ∼2,000-fold increases in miR-141 levels compared with the same cells infected with control (empty) vector (Supplementary Fig. 1j). Copy number measurement indicated that DU145 cells infected with Lenti-141 (Supplementary Fig. 1h) at an MOI of 0.5–50 resulted in only a slight increase in miR-141 copy numbers (Supplementary Fig. 1k). Remarkably, miR-141 overexpression via lenti-141 infection significantly suppressed xenograft tumour growth in CD44^+^ LAPC9 (Fig. 1h) and in bulk DU145 and PPC-1 (Fig. 1i,j) cells. A similar trend of tumour-inhibitory effect of miR-141 on CD44^+^ VCaP cells was also observed (Supplementary Fig. 2b). miR-141 suppression of PCa clonal and tumour growth was associated with inhibition of cell proliferation as evidenced by reduced 5-ethynyl-2′-deoxyuridine (EdU) incorporation in miR-141-overexpressing cells (Supplementary Fig. 2c–e) and reduced Ki-67^+^ cells in miR-141-overexpressing tumours (Supplementary Fig. 2f). miR-141 expression showed minimal effects on cell death and senescence (not shown). These results, together, suggest that miR-141 inhibits the regeneration and growth of both AR^+^ (LAPC9 and VCaP) and AR^−^ (DU145 and PPC-1) PCa, and that its PCa-suppressive functions are associated with inhibition of proliferation.

Of clinical relevance, lentiviral-mediated miR-141 overexpression inhibited clonal growth (Fig. 1k) and serial sphere formation in CD44^+^ (Fig. 1l,m) and bulk (Supplementary Fig. 2g) HPCa cells from patient tumours.

### miR-141 inhibits metastasis and exhibits therapeutic efficacy

**Figure 2:**
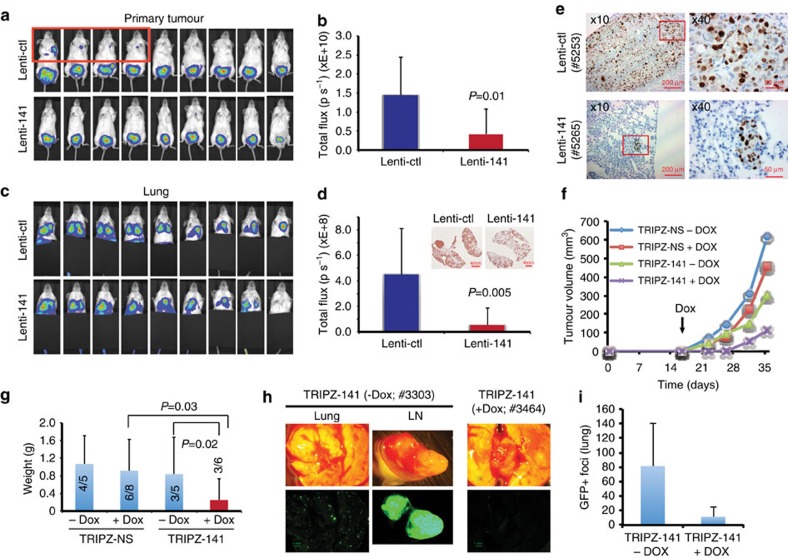
miR-141 inhibits lung metastasis of CD44^+^ PCa cells. (**a**–**d**) miR-141 inhibits lung metastasis of CD44^hi^ PC3 cells. Tumour growth and metastasis were monitored using bioluminescence imaging (IVIS). (**a**,**b**) Representative images (**a**) and quantification (**b**, in total photon flux) of primary tumours before termination of the experiment. (**c**,**d**) Representative images (**c**) and quantification (**d**) of lung metastasis with the primary tumours covered. In **a**, four animals showed lung metastasis without their primary tumours covered (in red box). Inserts in **d** are the representative GFP IHC images in the lungs (note significantly reduced GFP^+^ metastatic foci in the Lenti-141 lung). (**e**) miR-141 inhibits lung metastasis of CD44^+^ DU145 cells. Shown were representative IHC images of human-specific Ki-67 staining in mouse lung. (**f**–**i**) miR-141 inhibits LAPC9 metastasis. Tumour volumes were measured (**f**). Mice were terminated at day 40 and endpoint tumours were harvested and weighted (**g**; incidence indicated in bars). Lungs (and LN and some other organs) were harvested (**h**) to analyse metastasis by assessing GFP^+^ metastasis foci (**i**). Values represent mean±s.d., for **b**,**d** (*n*=9 for each group). For **g**, the animal # for each group was indicated by the denominators (that is, 5, 8, 5 and 6, respectively), For **i**, *n*=3 for each group. For images in **d** (insets), **e**,**h**, the original scale bars are indicated. *P* values were calculated using unpaired student's *t*-test.

The CD44^+^ PCa cell population acutely purified from xenograft models possesses high metastatic potential^13^,^20^,^25^; thus, we determined whether manipulating miR-141 levels in CD44^+^ or bulk PCa cells would affect their metastatic capabilities. To this end, we introduced miR-141 via lentiviral infection into luciferase (luc)-tagged CD44^hi^ PC3 cells, which expressed little endogenous miR-141 (Supplementary Fig. 1c), and implanted the cells into the dorsal prostate (DP) of non-obese diabetic/severe combined immunodeficiency (NOD/SCID) mice^13^,^20^,^25^. miR-141 overexpressing CD44^hi^ PC3 cells produced smaller primary tumours (Fig. 2a,b) and less lung metastasis (Fig. 2c,d). We also implanted the miR-141-overexpressing CD44^+^ DU145 cells into the DP of NOD/SCID mice and observed similar inhibitory effects of miR-141 on metastasis in the lungs (Fig. 2e and Supplementary Fig. 3a,b).

To test whether miR-141 might posses any ‘therapeutic' efficacy, we established a Doxycycline (Dox)-inducible miR-141 lentiviral expression system (pTRIPZ-141; Supplementary Fig. 3c), which only increased miR-141 levels in DU145 cells by ∼11–13-fold (Supplementary Fig. 3d). We implanted LAPC9-GFP cells infected with pTRIPZ-141 or control vector (pTRIPZ-NS) into the DP and allowed tumours to develop for ∼2 weeks to reach palpable sizes (∼2–3 mm diameter). We then induced miR-141 expression with Dox feed on day 16. We observed that miR-141 induction inhibited growth of LAPC9 xenografts (Fig. 2f–g and Supplementary Fig. 3e) and reduced lung metastasis (Fig. 2h,i and Supplementary Fig. 3f,g).

Collectively, these results indicate that miR-141 inhibits the metastasis in both AR^−^ (PC3 and DU145) and AR^+^ (LAPC9) PCa models.

### miR-141 inhibits PCa cell invasion and directly targets CD44

**Figure 3:**
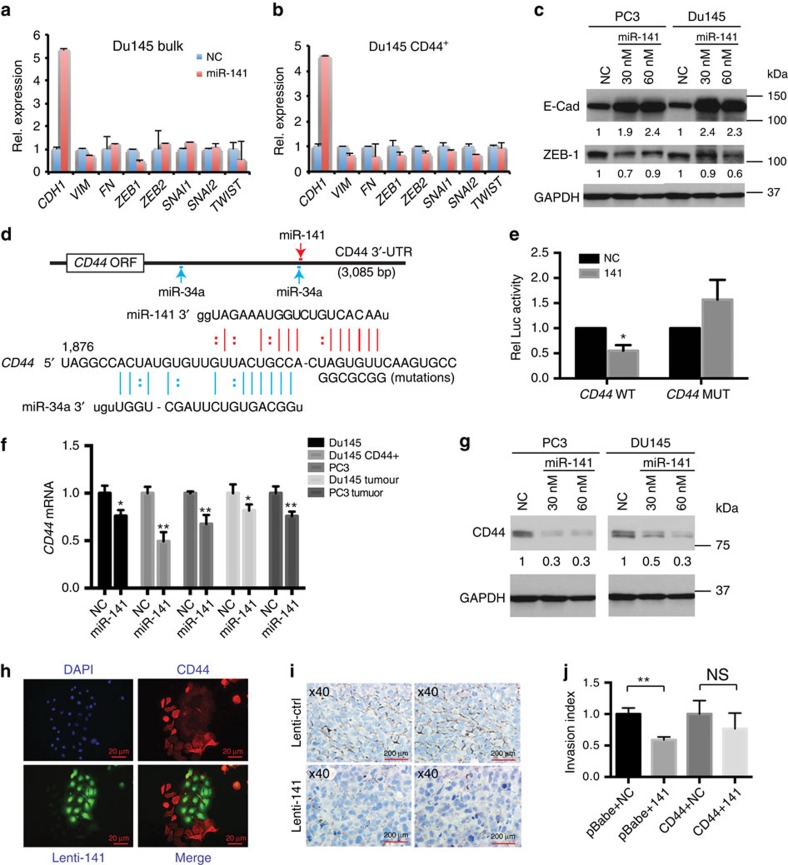
Partial suppression of EMT by miR-141 and CD44 as a direct target of miR-141. (**a**,**b**) qPCR analysis of EMT markers in bulk (**a**) or CD44^+^ (**b**) DU145 cells transfected with the miR-141 oligos. Results were presented as the relative expression levels over the cells transfected with the NC oligos. (**c**) WB of E-Cadherin and ZEB-1 in PC3 and DU145 cells transfected with NC (60 nM) or miR-141 (30 or 60 nM) oligos. GAPDH was used as control and the relative protein levels after normalizing to GAPDH and NC controls were indicated below each lane. (**d**) Schematic of predicted miR-141- and miR-34a-binding sites in the *CD44* 3′-UTR. Shown below is the actual sequence in the region with the mutated miR-141-binding sequence. (**e**) Luc reporter assays in DU145 cells cotransfected with either wild-type CD44 3′-UTR or mutated 3′-UTR construct with miR-141 or NC oligos. (**f**) qPCR analysis of *CD44* mRNA levels in the indicated PCa cells overexpressing miR-141 and in endpoint tumours generated from miR-141-overexpressing PCa cells. (**g**) Representative WB images of CD44 in PC3 and DU145 cells transfected with NC (60 nM) or miR-141 (30 and 60 nM) oligos harvested at 72 h. (**h**) IF staining of CD44 (red) in PC3 cells transduced with Lenti-141 (green) reveals a mutually exclusive expression pattern of CD44 and miR-141 (x200). Shown are representative images. (**i**) Representative CD44 IHC images in two pairs of endpoint DU145 tumours derived from cells initially transduced with Lenti-141 or Lenti-ctl. (**j**) Invasion assays in DU145 cells co-transfected with NC or miR-141 oligos together with pBabe empty vector or pBabe-CD44 vector. Values represent mean±s.d. of triplicates from three independent experiments. *P<0.05 and **P<0.01. *P* values were calculated using unpaired student's *t*-test.

To determine molecular mechanisms underlying the metastasis-suppressing effects of miR-141 in PCa cells, we performed Matrigel invasion assays, which revealed that miR-141 significantly inhibited invasive capabilities of several different types of PCa cells including AR^−^ (DU145 and PC3; Supplementary Fig. 4a,b), primary patient HPCa (Supplementary Fig. 4c,d) and AR^+^ (VCaP, LAPC4 and LNCaP C4-2; Supplementary Fig. 4e) PCa cells. The miR-200 family members, including miR-141, are well-established negative regulators of EMT^7^,^8^,^9^,^10^. As miR-141 inhibits PCa cell invasion and metastasis, we wondered whether miR-141 might be exerting its effects by suppressing EMT (Fig. 3a–c). We found that miR-141 expression in CD44^+^ DU145 cells caused significant increase in *E-cadherin* (*CDH1*) and generally reduced mRNA levels of mesenchymal markers including *VIM* and *ZEB1* (Fig. 3b). In bulk DU145 cells, miR-141 also led to increased *CDH1* and decreased *VIM* and *ZEB1* (Fig. 3a). Consistently, miR-141 increased CDH1 and reduced ZEB1 protein levels in bulk DU145 and PC3 cells (Fig. 3c). Interestingly, however, quantitative PCR (qPCR) analysis showed that in bulk DU145 cells, miR-141 did not reduce the mRNA levels of most other EMT markers including *FN*, *ZEB2*, *SNAI1*, *SNAI2* and *TWIST* (Fig. 3a). These latter observations suggest that miR-141 might be inducing a ‘partial' MET phenotype^27^,^28^,^34^,^35^,^36^ in PCa cells in that it induces a strong epithelial phenotype (evidenced by increased CDH1) but only a partial loss of mesenchymal genes. Recent studies have suggested that CSCs may not always be associated with a complete mesenchymal phenotype—rather, cancer cells with a partial EMT phenotype that allows a great proliferative capability of epithelial cells and the morphological plasticity of mesenchymal cells will have the best chance to survive and establish a tumour or metastatic colony^36^,^37^,^38^. Consistent with this notion, CD44^+^ PCa/HPCa cells, although possessing high clonogenic, tumour-regenerating and metastatic potentials^13^,^20^,^21^,^23^,^25^, did not manifest a ‘pure' mesenchymal gene expression profile (Supplementary Fig. 4f–j).

In our previous study^13^, miR-34a, which was under-expressed in CD44^+^ PCa/HPCa cells, was found to directly target *CD44* mRNA at two sites of the 3′-untranslated region (3′-UTR) (Fig. 3d). As miR-141 was also under-expressed in CD44^+^ PCa (Fig. 1a) and HPCa (Fig. 1c) cells, and there was a good inverse correlation between % CD44^+^ PCa cells and relative miR-141 levels (Fig. 1b), we suspected that miR-141 might also target *CD44*. Indeed, several miRNA target prediction programmes identified a putative miR-141 binding site in the 3′-UTR of *CD44* that partially overlapped with and localized just slightly downstream of the second miR-34a-binding site (Fig. 3d). Luc assays indicated that co-transfection of the luc reporter and miR-141 oligos into DU145 cells produced lower luc activities than cells co-transfected with the reporter construct plus NC oligos (Fig. 3e). Mutation of the miR-141-binding sequence abrogated the suppressive effect of miR-141 (Fig. 3e). Overexpression of miR-141 in bulk DU145 and PC3 cells or in CD44^+^ DU145 cells significantly reduced CD44 mRNA and protein levels (Fig. 3f,g). Notably, transfection in DU145 and PPC-1 cells with miR-141 mimics at 3 nM, which only minimally increased miR-141 levels (Supplementary Fig. 1g), significantly reduced *CD44* mRNA levels in these cells (Supplementary Fig. 5a,b; left). Infection of cells with low MOI of Lenti-141 (that is, 2.5 and 5) similarly reduced *CD44* mRNA levels (Supplementary Fig. 5a,b right). Immunofluorescence staining for CD44 in PC3 cells that had been transduced with Lenti-141 revealed a stunning contrasting pattern such that the miR-141-expressing (that is, GFP^+^) cells were largely devoid of CD44 expression (Fig. 3h). We also found that the endpoint tumours derived from miR-141-overexpressing PCa cells expressed lower levels of CD44 mRNA (Fig. 3f) and protein (Fig. 3i) than the control tumours. Finally, we performed functional ‘rescue' invasion assays by overexpressing a *CD44* complementary DNA^13^ lacking the miR-141 target site. As shown in Fig. 3j, miR-141 oligo transfection inhibited DU145 cell invasion, which was partially overcome by expressing exogenous *CD44*. Altogether, these studies (Fig. 3d–j) identify CD44 as a direct target of miR-141 in PCa cells.

### RNA-Seq analysis reveals novel pathways regulated by miR-141

**Figure 4:**
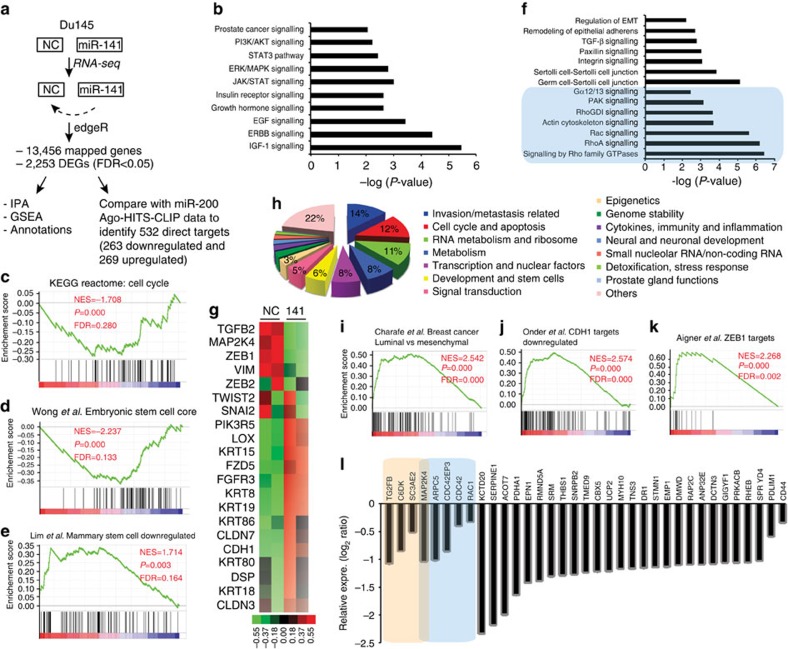
RNA-Seq analysis reveals novel miR-141 targeted signalling molecules and pathways. (**a**) Experimental scheme of RNA-Seq in DU145 cells and subsequent data analysis. (**b**) Major mitogenic pathways affected by miR-141 expression in DU145 cells revealed by IPA. (**c**–**e**) GSEA reveals negative enrichment of miR-141-altered genes in cell cycle (**c**) and ESC core (**d**) gene sets but positive association with ‘mammary stem cell downregulated' gene set (**e**). (**f**) IPA showing that miR-141 impacted several pathways involved in EMT and Rho family GTPase signalling (shaded). (**g**) Heat map of representative EMT-related molecules from DU145 RNA-Seq results.(**h**) Functional classification of the 949 genes downregulated by miR-141 in DU145 cells. The non-redundant functional classification was conducted as previously described^23^,^25^,^39^. The % for most functional categories is indicated. (**i**–**k**) GSEA reveals positive enrichment of miR-141-altered genes in luminal (that, differentiated epithelial) breast cancer cells (**i**), CDH1 targets downregulated (**j**) and ZEB1 targets (**k**) gene sets. See annotations for each data set in Methods. (**l**) The top 35 putative direct targets of miR-141 as revealed by merging DU145 RNA-Seq data with the miR-200 Ago-HITS-CLIP-Seq data^12^. Bars represent the relative expression levels of each gene from RNA-Seq.

To identify novel targets of miR-141, we performed genome-wide RNA-Seq in DU145 (Fig. 4) and LAPC9 (Supplementary Fig. 6) cells transfected with miR-141 and NC oligos. Of the 13,456 mapped genes in DU145 cells, we identified 2,253 differentially expressed genes (DEGs; false discovery rate (FDR)<0.05) including 949 down- and 1,304 upregulated genes (Fig. 4a and Supplementary Data 1). Ingenuity Pathway Analysis (IPA) in the 2,253 DEGs revealed ‘prostate cancer signalling' and several growth factor signalling pathways including PI3K/AKT, ERK/MAPK, IGF-1/insulin receptor and epidermal growth factor/ERBB, and growth hormone signalling among the pathways most significantly affected by miR-141 (Fig. 4b). These latter findings suggest that miR-141 may dampen mitogenic signalling to inhibit cell cycle progression, which would be consistent with our earlier observations that miR-141 suppresses cell proliferation (Supplementary Fig. 2c–f). In further support, Gene Set Enrichment Analysis (GSEA) revealed that the miR-141 gene expression profile was negatively associated with the KEGG REACTOME ‘CELL CYCLE' term (Fig. 4c). Moreover, the miR-141 DEGs were enriched in signal transducer and activator of transcription 3 (STAT3) and JAK/Stat3 signalling (Fig. 4b), a well-known regulator of stem cells, suggesting that miR-141 may negatively have an impact on (cancer) stem cell signalling in DU145 cells. In support, GSEA demonstrated that the miR-141-induced gene expression changes negatively correlated with embryonic stem cells (ESC) core genes (Fig. 4d) and positively correlated with ‘mammary stem cell downregulated' (Fig. 4e). Together, these analyses indicate that miR-141 targets genes that normally promote cell cycle progression and stem cell activities, consistent with our preceding biological studies.

RNA-Seq data also provided strong support to our earlier observations that miR-141 inhibits PCa cell invasion and metastasis (Fig. 4f–k). For instance, functional annotations^23^,^25^,^39^ of the miR-141 downregulated 949 genes in DU145 cells revealed ‘invasion/metastasis' and ‘cell cycle' to be the two major classes of the affected genes (Fig. 4h). Furthermore, consistent with well-established regulation of EMT by miR-200 family^7^,^8^,^9^,^10^, miR-141 caused changes in genes involved in EMT, transforming growth factor-β, cell–cell junction and cytoskeleton, and other related signalling processes (Fig. 4f). Many epithelial markers including E-cadherin, Claudins (for example, CLDN7 and CLDN3) and cytokeratins (KRT) were strongly induced by miR-141 (Fig. 4g). GSEA also showed that the miR-141 gene profile was strongly enriched in normal prostatic luminal epithelial cells we recently reported^39^ (normalized enrichment score (NES)=1.87; *P*=0.000; FDR=0.000). Meanwhile, miR-141 downregulated *TGFB2*, *ZEB1*, *MAP2K4* and *VIM* but caused inconsistent changes in *ZEB2*, *TWIST2* and *SNAI2* (Fig. 4g). These results indicate, again, that miR-141 induces a strong epithelial phenotype but only a partial loss of mesenchymal gene expression (Fig. 4i–k).

Strikingly, we observed, among the miR-141 downregulated genes, a strong over-representation of genes associated with cytoskeleton remodelling and cell motility including Rho GTPases, as well as its upstream regulators and downstream effectors (Fig. 4f, shaded). A recent study using Ago-HITS-CLIP approach identified a role of miR-200 family targets in coordinated control of actin cytoskeleton dynamics^12^. As the miR-200 members share largely overlapping downstream targets^8^,^9^,^10^, we took advantage of this argonaute high throughput sequencing-crosslinking immunoprecipitation (CLIP-Seq) data that provided direct target association information of miR-200a, which shares identical seed sequence with miR-141, and compared it with our RNA-seq data to identify potential targets of miR-141 in PCa cells. From the 2,253 DEGs, 532 transcripts (263 down- and 269 upregulated) were found to have binding sites to miR-200a/miR-141 (Fig. 4a, Supplementary Fig. 6 and Supplementary Data 2). Among the top 35 downregulated ‘direct' target genes were 4 known miR-141 targets (that is, *TGFB2*, *CDK6*, *SEC23A* and *MAP2K4*), 5 genes involved in Rho GTPase signalling (*MAP2K4*, *ARPC5*, *CDC42EP3*, *CDC42* and *RAC1*) and *CD44* (Fig. 4l and Supplementary Fig. 6).

An RNA-Seq experiment in xenograft LAPC9 cells overexpressing miR-141 also revealed that: (1) miR-141 significantly downregulated metastasis-associated genes; (2) miR-141 impacted actin cytoskeleton and Rho GTPase signalling; (3) miR-141 upregulated *CDH1* and many epithelial genes, whereas downregulated numerous mesenchymal genes; and (4) miR-141 directly targeted ∼150 genes, many of which were shared with the predicted targets in DU145 cells (Supplementary Figs 6 and 7a–h, Supplementary Data 3 and 4, and Supplementary Note 1).

### Rho GTPase signalling components as direct targets of miR-141

The Rho family of small GTPases plays critical roles in regulating actin dynamics, organelle development, cell movement and other cellular functions^40^,^41^,^42^. Three major Rho GTPases, RhoA, RAC1 and CDC42, regulate different yet intertwined aspects of cell movement: RhoA mainly modulates (actin) stress fibres and focal adhesion formation, and RAC1 mainly regulates lamellipodia via ARP2/3 complex, whereas CDC42 is not only a major inducer of filopodia but also can activate ARP2/3 complex to form invadosomes. ARPC5 encodes one of the seven subunits of human ARP2/3 complex and is a major regulator of actin cytoskeleton. CDC42EP3 encodes the effector protein downstream of CDC42, which is involved in actin cytoskeleton re-organization during cell shape changes including pseudopodia formation.

**Figure 5:**
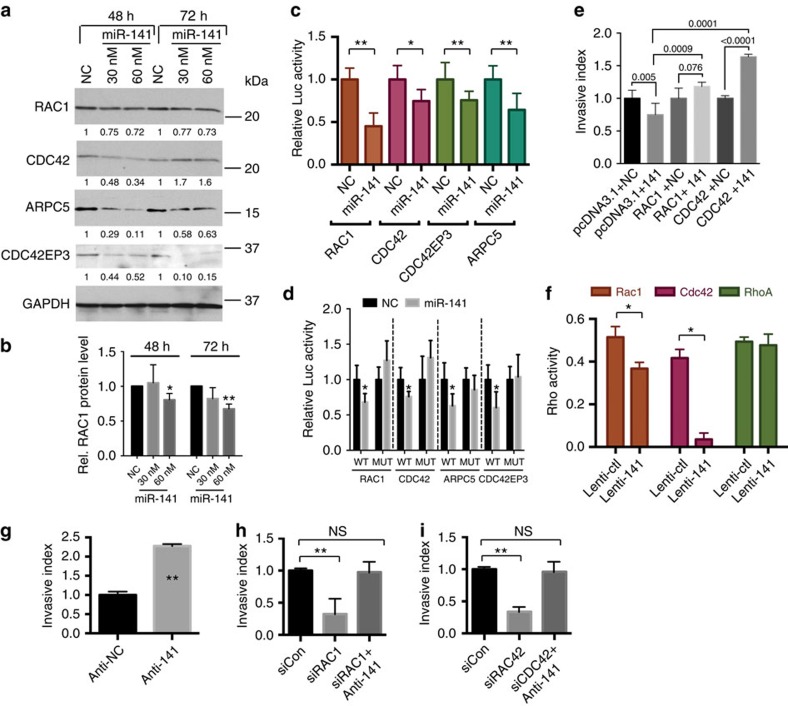
Rho GTPase signalling pathway members are direct targets of miR-141. (**a**) WB of RAC1, CDC42, ARPC5 and CDC42EP3 in DU145 cells transfected with NC or miR-141 oligos at the two doses and time intervals. Indicated below are relative expression levels (normalized to respective GAPDH and compared to NC) of each protein determined by densitometric scanning of the individual bands. (**b**) Bar graph of relative protein level of RAC1. Values represent mean±s.d. from four independent experiments. *P<0.05 and **P<0.01. (**c**) Luc reporter assays in DU145 cells co-transfected with wild-type RAC1, CDC42, ARPC5 or CDC42EP3 3′-UTRs and 30 nM miR-141 or NC oligos. Values represent mean±s.d. (*n*=4). *P<0.05 and **P<0.01. (**d**) Luc reporter assays in DU145 cells co-transfected with wild-type or mutant RAC1, CDC42, ARPC5 or CDC42EP3 3-′UTRs together with NC or miR-141 oligos (30 nM) for 72 h. Values represent mean±s.d. (*n*=4). *P<0.05. (**e**) Invasion assays in DU145 cells co-transfected with NC or miR-141 oligos together with pcDNA3.1 empty vector or pcDNA-RAC1 or pcDNA-CDC42 vector. Values represent mean±s.d. from triplicates in three independent experiments, *P*-values are indicated above. (**f**) Rho GTPase activities were measured in DU145 cells infected with Lenti-141 or control lentivirus. Values represent mean±s.d. of duplicates from three independent experiments. *P<0.05. (**g**–**i**) Invasion assays in DU145 cells transfected with the antisense oligos to NC or miR-141 (anti-141) (**g**), or transfected with 10 nM of scramble siRNA control (siCon) or siRNAs targeting RAC1 (**h**), or CDC42 (**i**), either alone or in combination of anti-141. Values represent mean±s.d. of triplicates from three independent experiments.*P<0.05 and **P<0.01. *P* values were determined using unpaired student's *t*-test.

As our RNA-Seq analysis implicated RAC1, CDC42, CDC42EP3 and ARPC5 as direct miR-141 targets in PCa cells (Fig. 4l and Supplementary Fig. 7a–h), we performed western blotting (WB) analysis to determine the impact of miR-141 on the protein levels of these four molecules. miR-141 downregulated, to varying degrees, the four proteins (Fig. 5a,b). Notably, miR-141 mimics at low concentrations (that is, 3 and 15 nM) and Lenti-141 at low MOI (for example, 5) also downregulated the mRNA levels of *RAC1* and *CDC42* in PCa cells (Supplementary Fig. 7i). Luc reporter assays indicated that miR-141 inhibited the luc activities of the constructs containing the 3′-UTR of *RAC1*, *CDC42*, *CDC42EP3* or *ARPC5* (Fig. 5c). Importantly, site-specific mutagenesis of the miR-141-binding sites abrogated the miR-141 suppression of the reporter activities (Fig. 5d), confirming direct miR-141 binding to these Rho GTPases. To further validate that these molecules represent the functional mediators of miR-141 effects, we performed ‘rescue' invasion experiments, in which we overexpressed *RAC1* and *CDC42* coding constructs that lacked the miR-141 binding site(s) at the 3′-UTRs. As shown in Fig. 5e, overexpression of miR-141 inhibited DU145 cell invasion, but the inhibitory effect was overcome by the expression of non-targetable *RAC1* or *CDC42* cDNA. Furthermore, miR-141 overexpression suppressed the GTPase activities of RAC1 and CDC42 but not RhoA (Fig. 5f) and, interestingly, RhoA was not predicted to be a direct miR-141 target. Of note, infection of PCa cells with Lenti-141 at low MOI (that is, 2.5 and 5) also decreased the Rho GTPase activities of both RAC1 and CDC42 (Supplementary Fig. 7j). Finally, the antisense oligos of miR-141 (that is, anti-141), when transfected into PCa cells, promoted invasion (Fig. 5g). Importantly, small interfering RNA (siRNA)-mediated silencing of RAC1 or CDC42 inhibited PCa cell invasion, which was ‘rescued' by co-transfection of anti-141 (Fig. 5h,i). These results, collectively, suggest that miR-141 regulates PCa cell motility/invasion via suppressing several members of Rho GTPase signalling pathway.

### EZH2 represents yet another novel target of miR-141

**Figure 6:**
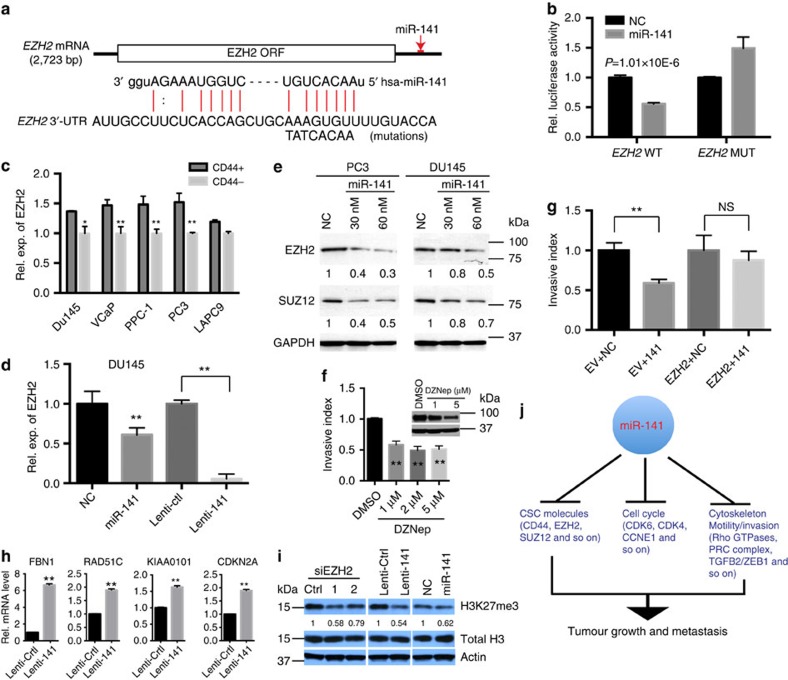
EZH2 is a direct target of miR-141. (**a**) Putative binding site of miR-141 in *EZH2* 3′-UTR. (**b**) Luc reporter assays in DU145 cells co-transfected with either wild-type EZH2 3′-UTR or the 3′-UTR with the predicted miR-141-binding site mutated and miR-141 or control oligos. (**c**) qPCR analysis of *EZH2* mRNA levels in CD44^+^ PCa cells. (**d**) qPCR of EZH2 in Du145 cells overexpressing miR-141 using either oligo transfection or lentiviral infection. (**e**) WB of EZH2 and SUZ12 in PC3 and Du145 cells overexpressing miR-141. Relative protein levels were measured by densitometry and normalized to GAPDH levels (indicated below). (**f**) Invasion assay in DU145 cells treated with the EZH2 inhibitor DZNep. Inset, WB showing reduced EZH2 proteins by DZNep. (**g**) Invasion assays in Du145 cells co-transfected with 30 nM NC or miR-141 oligos together with empty vector or pCMV-EZH2 construct. (**h**) qPCR of EZH2 target genes including *FBN1*, *RAD51C*, *KIAA0101* and *CDKN2A* in DU145 cells infected with lenti-Ctrl or lenti-141. (**i**) WB analysis of H3K27me3 in DU145 cells overexpressing miR-141 using either lentiviral vectors or oligo transfection. Total H3 and actin were included as endogenous controls. (**j**) miR-141 exhibits pleiotropic anti-tumour and anti-metastasis effects in PCa by targeting multiple molecules associated with CSCs, cell cycle and proliferation, and cytoskeleton, cell motility/invasion and metastasis. Values represent mean±s.d. of triplicates from three to five independent experiments. *P<0.05 and **P<0.01. *P* values were determined using unpaired student's *t*-test.

Interestingly, IPA upstream regulator analysis implicated EZH2, a histone methyltransferase and a critical component of the PRC2 (polycomb repressive complex 2) that is frequently overexpressed in aggressive forms of prostate and other cancers, and involved in CSC maintenance, invasion and metastasis^43^,^44^,^45^,^46^, as a miR-141 ‘upstream regulator' (*P*=2.46 × 10^−7^), suggesting that EZH2 might be one of the mediators in miR-141-elicited global gene changes. In support, miR-141-induced gene expression profile was associated with ‘EZH2 Targets DN' in several data sets (Supplementary Fig. 6f). We observed a putative miR-141-binding site in the 3′-UTR of *EZH2* mRNA (Fig. 6a) and luc reporter assays showed that miR-141 suppressed the luc activities of the wild type but not mutated *EZH2* 3′-UTR (Fig. 6b). In addition, the CD44^+^ PCa cells generally expressed higher levels of *EZH2* mRNA (Fig. 6c) and miR-141 overexpression significantly reduced both RNA and protein levels of EZH2 in PCa cells (Fig. 6d,e). Interestingly, miR-141 expression also reduced the protein levels of SUZ12, another component of the PRC2 complex (Fig. 6e); SUZ12 (repressed) targets were enriched in our miR-141downregulated genes (Supplementary Fig. 8a) and we observed one putative miR-141-binding site at the 3′-UTR of SUZ12 using miRNA target prediction database (MicroRNA.org). These observations, overall, suggest that EZH2 (and perhaps SUZ12) is a direct target of miR-141. Consistent with this suggestion, 3-Deazaneplanocin A hydrochloride, a small-molecule EZH2 inhibitor, reduced EZH2 protein and blocked DU145 cell invasion (Fig. 6f). EZH2 knockdown (Supplementary Fig. 8b) also inhibited PCa cell invasion, which was alleviated by anti-141 (Supplementary Fig. 8c). Significantly, miR-141 inhibition of PCa cell invasion could be partially relieved by overexpressing an exogenous *EZH2* cDNA that lacked the miR-141-targeting sequence (Fig. 6g). Importantly, we interrogated the functional relationship between miR-141 and EZH2. As expected, EZH2 knockdown led to de-repression of four EZH2-repressed genes (*FBN1*, *KIAA0101*, *RAD51C* and *CDKN2A*)^44^,^45^ (Supplementary Fig. 8d). Remarkably, miR-141 similarly de-repressed the expression of 4 EZH2 targets (Fig. 6h) and decreased levels of H3K27me3 (Fig. 6i). These results, altogether, provide convincing evidence that EZH2 represents a functionally relevant target of miR-141 in PCa cells.

## Discussion

The present study represents the most comprehensive investigations on the role of miR-141 in regulating both a defined PCa cell population, that is, CD44^+^, and bulk PCa cells. CD44 has been employed to enrich CSCs in multiple cancers^2^,^13^. In PCa, high CD44 expression alone or in combination with other markers such as ALDH and integrin α2β1 enriches for tumorigenic and metastatic PCa cells^13^,^20^,^21^,^23^,^25^. CD44 is not only a phenotypic marker for PCSCs but also functionally important for PCa cells to manifest their metastatic potential^13^,^25^. miR-141 was initially identified to be one of the four miRNAs commonly under-expressed in CD44^+^, CD133^+^, and α2β1^+^ PCa populations^13^,^14^. Among the other 3 under-expressed miRNAs, that is, miR-34a, Let-7b and miR-106a, both miR-34a and Let-7b possess strong tumour- and/or metastasis-suppressive functions^13^,^14^. By first confirming the under-expression of miR-141 in PCa stem/progenitor populations, here we show that miR-141 is most highly under-expressed (compared with the other three miRNAs) in patient tumour-derived CD44^+^ HPCa cells (Fig. 1c, 14). miR-141 re-expression inhibits clonal and clonogenic properties in CD44^+^ and bulk PCa/HPCa cells *in vitro*, as well as tumour regeneration in four xenograft models. The tumour-suppressive functions of miR-141 are associated with inhibition of cell proliferation, as evidenced by EdU and Ki-67 staining, and supported by RNA-Seq data (Fig. 6j). Of interest, a side-by-side comparison in DU145 cells by analysing the impact of overexpressing miR-34a, Let-7b and miR-141 on cell growth and invasion revealed that miR-34a demonstrated the strongest inhibitory effects, whereas miR-141 and Let-7b showed overall similar inhibitory effects (Supplementary Fig. 8e).

One of the most important findings here is that miR-141, similar to miR-34a^13^, exhibits PCa metastasis-suppressing effects, as observed in three orthotopic models (that is, PC3, DU145 and LAPC9) in which miR-141 overexpressing PCa cells produced less metastasis. Interestingly, pilot studies with tail vein-injected cells revealed that mice with the prostate-implanted miR-141-expressing PCa cells showed less circulating GFP^+^ HPCa cells in the peripheral blood than the animals that received control PCa cells (Supplementary Fig. 8f), suggesting that miR-141 probably suppresses the metastatic cascade at an early stage. Consistently, miR-141 enforced a strong epithelial phenotype by upregulating E-cadherin, cytokeratins and many other epithelial markers as demonstrated by both qPCR and RNA-Seq analyses. Consequently, miR-141 inhibits cell motility and invasion. Interestingly, although miR-200 family members are well-known suppressors of EMT, miR-141 only induces a partial loss of EMT in PCa cells by consistently suppressing ZEB1 and VIM but not most other mesenchymal genes.

At the molecular level, in addition to repressing ZEB1 and VIM, and upregulating E-cadherin, miR-141 also impacts many other novel targets. Similar to miR-34a^13^, miR-141 directly binds to and downregulates CD44 (Fig. 6j). Intriguingly, the miR-141-binding site at the *CD44* 3′-UTR juxtaposes and partially overlaps with the distal miR-34a-binding site (Fig. 3d). Comparative *CD44* 3′-UTR luc studies show that miR-34a induces a much stronger inhibition of luc activity than miR-141 (Liu C *et al*., unpublished). Notably, several other miRNAs including miR-708 and miR-199a-3p also directly targets CD44 to regulate PCSC activity and PCa development^47^,^48^. Simultaneous regulation of CD44 by at least four validated miRNAs (that is, miR-34a, miR-708, miR-199a-3p and miR-141) highlights the importance in tightly controlling its expression and also explains the deficiency of these miRNAs in CD44^+^ PCa cells. Another significant finding in this study is that in addition to CD44, miR-141 directly targets several Rho GTPases and also EZH2 (Fig. 6j). As the Rho GTPases play critical roles in controlling actin cytoskeleton, cell motility and invasion, these novel findings further suggest that miR-141 negatively regulates early metastatic events. EZH2 has been shown to be oncogenic in PCa via regulating cell invasion and CSC activities^43^,^44^. Of interest, PRC2 epigenetically represses the expression of miRNAs including miR-200, miR-181 and miR-203 (ref. 49). These observations, together with miR-141 directly targeting EZH2, suggest a regulatory circuitry between miRNAs and the PRC2 epigenetic silencing machinery.

Some functional studies herein were conducted by transfecting PCa cells with 30 nM synthetic miR-141 oligos and miRNA mimic transfection has been shown to cause ‘supraphysiological' levels of intracellular miRNA and accumulation of artifactual RNA species^50^. Indeed, transfection of 30 nM of miR-141 in PCa cells can, in a cell type-dependent manner, result in >100–67,000-fold increase in miR-141 levels compared with NC transfected cells. On the other hand, measurement of copy numbers in 20 freshly purified patient tumour-derived HPCa cells, surprisingly, reveals a wide range of ‘physiological' levels of endogenous miR-141, from undetectable to 103,800 copies per cell (Supplementary Fig. 1d,e). Even cultured and xenograft-derived PCa cells exhibit a wide variation in endogenous miR-141 copy numbers. These findings suggest that PCa cells *in vitro* and *in vivo* express a range of, rather than a set, ‘physiological' levels of miR-141. Notably, 3 nM of miR-141 mimics, which did not lead to a dramatic increase in intracellular miR-141, also significantly reduced the mRNA levels of *CD44* (Supplementary 5a,b) and *RAC1* (Supplementary Fig. 7i). Importantly, we performed and validated many of our biological studies using lentiviral-mediated overexpression, which does not cause supraphysiological levels of miRNA or accumulation of artefactual RNA species^50^. Thus, constitutive lentiviral-mediated miR-141 expression, which led to a slight increase in miR-141 copy numbers, impressively inhibited clonal and clonogenic capacities of primary HPCa cells (Fig. 1k–m), xenograft tumour regeneration and growth (Fig. 1h–j and Supplementary Fig. 2b), and metastasis (Fig. 2a–e and Supplementary Fig. 3a,b). Furthermore, Dox-inducible lentiviral miR-141 expression caused only 11–13-fold increases in miR-141 levels but greatly inhibited metastasis in several PCa models (Fig. 2f–i and Supplementary Fig. 3c–g). Finally, Lenti-141, at low MOI (that is, 2.5 and 5), inhibited the mRNA levels of *CD44* (Supplementary Fig. 5a,b) and the Rho GTPase activities of RAC1 and CDC42 (Supplementary Fig. 7k). Collectively, numerous complimentary and cross-validating approaches we took in our studies deliver a clear message that miR-141 possesses prostate tumour and metastasis suppressive functions via targeting critical molecules including CD44, Rho GTPases and EZH2. One caveat to note is that the original CLIP-Seq experiment was performed in breast cancer cells^12^. Although miR-141 shares identical target seed sequence with miR-200a and we have experimentally and functionally validated a few deduced targets (for example, CD44, RAC1, ARPC5, CDC42 and CDC42EP3), ideally, miR-141-specific CLIP-Seq should be conducted in PCa cells.

Previous miRNA expression profiling studies in prostate tumour tissues and (unfractionated) bulk PCa cells have revealed conflicting results, that is, no change^51^,^52^,^53^, downregulation^54^,^55^,^56^, upregulation^57^,^58^,^59^ or cell type-dependent changes^60^, regarding miR-141 levels in PCa compared with normal/benign (N/B) tissues. Serum levels of miR-141 have been reported to be elevated in advanced PCa patients^30^. We find that prostate tumours in the TCGA PRAD database express significantly higher levels of miR-141 mRNA than neighboring N/B tissues (Supplementary Fig. 8g). Our own copy number measurements in 11 HPCa and 7 N/B samples (Supplementary Table 4) also revealed higher miR-141 levels in tumours (Supplementary Fig. 8h). Pilot *in situ* hybridization studies in three pairs of PCa and matching N/B tissues similarly revealed higher levels of miR-141 in tumours (Supplementary Fig. 8i). These results may appear seemingly contradictory to our observations here that miR-141 is under-expressed in tumorigenic PCa cells and miR-141 possesses tumour- and metastasis-suppressive functions. There are several possible explanations. First, PCSCs in untreated tumours always represent a minor cell population^24^,^25^,^26^,^61^. As miR-141 is predominantly expressed in the non-CSCs, which represent the bulk of the tumour, the miR-141 expression levels are thus expected to be higher in tumours. Second, PCa cells frequently have increased AR expression and/or AR activity, and there has been some evidence for cross-regulations of miR-141 and androgen signalling^58^,^60^. Indeed, we find that AR^+^ PCa cell lines generally express higher levels of miR-141 than AR^−^ PCa lines (Fig. 1b) and there exist several putative AR-binding sites in *miR-141* upstream genomic region (Supplementary Fig. 8j). Therefore, higher levels of miR-141 in prostate tumours could be partially related to increased AR expression/activity, consistent with the findings that PCSCs generally have lower AR expression^22^,^23^,^24^,^25^,^26^,^61^ (thus lower miR-141). Finally, higher miR-141 levels in the sera/plasma of advanced PCa patients^30^ are probably related to the disintegration of the prostate organ integrity, leading to the leaking of PCa cells to the blood stream. This is analogous to reduced prostate-specific antigen (PSA) mRNA/protein expression in PCSCs^23^,^25^ but significantly increased serum PSA levels in PCa patients.

In summary, the present study demonstrates that miR-141, devoid in PCSCs, suppresses prostate tumour growth and metastasis by targeting a cohort of prometastasis genes including *CD44*, *EZH2* and *Rho GTPases* (Fig. 6j). Coupled with our earlier studies on miRNA regulation of PCSCs^13^,^14^, the results herein reinforce the concept that lack of expression of several key tumour/metastasis suppressive miRNAs concertedly and coordinately confers CSC properties^4^. Our observations provide further rationale for developing these CSC-targeting miRNAs into novel, combinatorial anti-tumour and anti-metastasis replacement therapeutics^61^, as illustrated by miR-34a that is now in clinical trials^62^,^63^.

## Methods

### Cell lines and animals

DU145, LNCaP, PC3, PPC-1 and VCaP cells were obtained from American Type Cell Culture and cultured in RPMI-1640 (Life Technologies, Carlsbad, CA) plus 7% fetal bovine serum (FBS) with the exception of VCaP, which were cultured in DMEM medium (Life Technologies) supplemented with 15% FBS. Human xenograft prostate tumours, LAPC9 (bone metastasis; positive for androgen receptor (AR) and PSA), LAPC4 (lymph node metastasis; AR^+^ and PSA^+^) and DU145 (brain metastasis; AR^−^ and PSA^−^) were maintained in NOD/SCID mice. These cell and xenograft lines have been routinely used in our lab^13^,^14^,^18^,^20^,^21^,^23^,^25^,^33^, regularly authenticated by our institutional CCSG Cell Line Characterization Core using short tandem repeat analysis and checked to be free of mycoplasma contamination using the Agilent (Santa Clara, CA) MycoSensor QPCR Assay Kit (catalogue number 302107). NOD/SCID mice were produced mostly from our own breeding colonies with occasional purchases from the Jackson Laboratories and maintained in standard conditions according to the Institutional Guidelines. All animal experiments were approved by our Institutional Animal Care and Use Committee.

### RNA isolation and qPCR analysi**s**

Total RNA was extracted using miRNA isolation kit (Life Technologies) according to the manufacturer's instructions. miRNA qPCRs were performed using TaqMan miRNA assays (Life Technologies). qPCR data for miRNAs were normalized to RNU48, whereas qPCR data for mRNAs were normalized to GAPDH. Primer information was listed in Supplementary Table 1.

### Determination of miR-141 copy numbers

Copy numbers of miR-141 per cell in PCa cells or tissues were estimated using a qRT–PCR method, which contained a stem-loop RT followed by real-time PCR. We first used synthetic miR-141 over several orders to construct a standard curve, in which the stem-loop RT primers were annealed to miR-141 and extended in the presence of reverse transcriptase (TaqMan MicroRNA Reverse Transcription Kit, catalogue number 4366596, Ambion). Next, miR141-specific forward primer, TaqMan probe and reverse primer (Taqman MicroRNA Assay (has-miR-141-3p); calagoue number 4427975) were used for PCR reactions (Taqman Universal Master Mix II, calagoue number 44400040). Quantification of miRNAs was estimated based on measured *C*_T_ values. Finally, copy numbers per cell were estimated based on standard curve of miR-141 synthetic miRNA assuming 15 pg total RNA per cell.

### Primary HPCa processing

All HPCa samples used in this study (Supplementary Table 2) were obtained from PCa patients undergoing Da Vinci based radical prostatectomy with the written informed patient consent in accordance with federal and institutional guidelines and with the approved Institutional review Board (IRB) protocol (MDACC LAB04-0498). The protocol for processing HPCa samples to obtain high purity epithelial cancer cells was previously described^13^.

### WB analysis

WB analysis was routinely performed using primary antibodies listed in Supplementary Table 3; ECL mouse IgG, horseradish peroxidase-linked whole antibody (NA931V, GE Healthcare Life Sciences, Pittsburg, PA) or ECL rabbit IgG and horseradish peroxidase-linked whole antibody (NA934V, GE Healthcare Life Sciences). Several representative WB full films are presented in Supplementary Fig. 9.

### Transient transfection with oligonucleotides

PCa cells were transfected with 30 nmol l^−1^ of miR-141 mirVana mimics or non-targeting negative control (NC) oligos, or the Anti-miR-141 and Anti-NC antisense oligos (Life Technologies) using Lipofectamine RNAiMax (Life Technologies) per the manufacturer's instructions. After culturing for 48 h, transfected cells were harvested for *in vitro* and *in vivo* studies.

### Lentiviral-mediated overexpression of miR-141

Three different lentiviral vectors over-expressing miR-141 were generated in our lab, that is, Lenti-miR-141 (based on Lenti-miR over-expression system from System Biosciences, Mountain View, CA; Supplementary Fig. 1b), pGIPZ-miR-141 (based on pGIPZ lentiviral backbone from Open Biosystems, pGIPZ-NS as the control; Supplementary Fig. 1c) and pTRIPZ-141 (based on pTRIPZ-inducible lentiviral backbone from Open Biosystems, pTRIPZ-NS as the control; Supplementary Fig. 2d). pGIPZ and pTRIPZ lentiviral vectors were produced in HEK293T packaging cells with *Trans*-Lentiviral packaging system (Open Biosystems). Lenti-miR-141 and Lenti-ctl viruses were produced in 293T packaging cells with the packaging plasmid mix as previously described^13^. Titres were determined by infecting and counting the GFP^+^ 293T cells. PCa cells (from either cultures or xenografts) were infected with the lentiviral supernatant at various MOI in the presence of 8 μl ml^−1^ polybrene and harvested 48–72 h after infection for experiments.

### Clonal and sphere formation assays

For clonal assays, cultured PCa or HPCa cells freshly purified from patient primary tumours were plated at a clonal density (that is, 100 cells per well) in a six-well plate. The number of holoclones^33^ was counted several days later. For Matrigel-based sphere formation assays, cells in medium were plated (generally 1,000 cells per well) in Matrigel at 1:1 ratio in a total of 100 μl in 96-well plates. Spheres were enumerated 1–2 weeks after plating. For floating sphere formation assays, cells were plated in serum-free epithelial basal medium supplemented with B27 (Invitrogen) and 20 ng ml^−1^ epidermal growth factor and basic fibroblast growth factor in ultra-low attachment plate. Floating spheres that arose in 1–2 weeks were counted. For all these experiments, a minimum of triplicate wells was run for each condition and repeat experiments were performed when necessary and feasible.

### Migration and invasion assays

For Boyden Chamber invasion assays, we employed Biocoat Matrigel Invasion Chamber (BD, Franklin Lakes, NJ) following the manufacturer's instructions. Briefly, 100,000–200,000 of PCa cells after transfection with NC/miR-141 oligos or infection with miR-141 overexpressing lentivirus were seeded into each well. Medium with 20% FBS was used in the lower chamber as chemo-attractant. After 20 h, cells were fixed and stained using Hema 3 staining kit (Fisher Scientific). Representative images were taken for each membrane and cells were counted. Migration assays were simultaneously performed in control wells using identical protocol with Transwell (Costar; 8 μl PET) without Matrigel. In some experiments, DU145 cells were plated 1 day before and treated with 1, 2 and 5 μM of 3-Deazaneplanocin A for 96 h after which cells were harvested and used in invasion assays and WB analysis.

### EdU flow cytometry

EdU is a nucleoside analogue to thymidine and incorporated into DNA during active DNA synthesis. EdU incorporation assays were performed using Click-iT EdU Flow Cytometer Assays kit (C10418, Invitrogen) as per the manufacturer's instructions. Briefly, PPC-1 bulk cells or CD44^+^ DU145 cells transfected with NC or miR-141 oligos (30 nM) for 48 h were pulsed with 10 μM EdU for 2.5 h and harvested. After fixation and permeabilization, cells were processed for EdU immunostaining and also labelled with Pacific Blue azide (1:200) and finally analysed on a flow cytometer.

### Luc reporter assays

The human *CD44* 3′-UTR was amplified from DU145 genomic DNA using primers 5′-AGAGCTCCACCTACACCATTATCTTG-3′ and 5′-TAAGCTTGGAAGTCTTCAGGAGACAC-3′, as previously decribed^13^. For site-specific mutagenesis, the region in the *CD44* 3′-UTR complementary to the seed sequence of miR-141 were mutated (5′-TAGTGTT-3′ to 5′-GGCGCGG-3′; see Fig. 3d). The human *EZH2* 3′-UTR was amplified from DU145 genomic DNA using primers 5′-GAGCTC-CATCTGCTACCTCCTCCCC-3′ and 5′-AAGCTTGACAAGTTCAAGTATTCTTTATT-3′. For site-specific mutagenesis, the region complementary to the seed sequence of miR-141 was mutated (5′-AAAGTGTT-3′ to 5′-TATCACAA-3′; see Fig. 6a). The PCR fragments were cloned into pGEM-T vector (Promega, Madison, WI) and sequence confirmed. Cells were plated in 24-well plates and co-transfected, using Lipofectamine 2000, with 1 μg firefly luc reporter plasmid and 5 ng *Renilla* plasmid (phRL-CMV) into which the 3′-UTR fragments were cloned. The luc activities were detected using Duo-luc reporter assay system (Promega). Both wild and mutant human RAC1 (5′-GTGTT-3′ to 5′-CACAA-3′; see Supplementary Fig. 5f), CDC42 (5′-GTGTT-3′ to 5′-CACAA-3′; see Supplementary Fig. 5e), CDC42EP3 (5′-GTT-3′ to 5′-CAA-3′; see Supplementary Fig. 5d) and ARPC5 (5′-GTGTT-3′ to 5′-CACAA-3′; see Supplementary Fig. 5c) 3′-UTR luc reporter constructs were obtained from SwitchGear Genomics (Menlo Park, CA) and luc activities were measured as per the manufacturer's instructions using LightSwitch luc assay kit.

### Rho family GTPase activity assay

The activity of Rho family of small GTPases including RhoA, RAC1 and CDC42 were detected using G-LISA small G-protein activation assays as per the manufacturer's instructions (Cytoskeleton, Inc., Denver, CO; catalogue number BK135).

### RNA-Seq and bioinformatics

DU145 and LAPC9 cells were transfected with 30 nM of miR-141 or NC oligos for 48 h. Total RNA was purified using RNeasy mini kit (Qiagen, Hilden, Germany). We first validated the overexpression of miR-141 by qPCR. One hundred nanograms of total RNA samples was then converted to cDNA using a NuGEN Ovation RNA-Seq System v2 according to the manufacturer's protocol (NuGEN, San Carlos, CA). NuGEN-amplified double-stranded cDNAs were fragmented into ∼180 bp using a Covaris system (Covaris, Woburn, MA). Fragmented cDNAs were run on a SPRI-TE library construction system (Beckman Coulter, Fullerton, CA) and during the adaptor ligation step, uniquely indexed NEXTflex adapters (Bioo Scientific, Austin, TX) were used for each of the samples to allow for multiplexing. Adapter-ligated libraries were enriched by PCR using a KAPA library amplification kit (KAPA Biosystems, Wilmington, MA) (1 cycle at 98 °C for 45 s; 7 cycles at 98 °C for 15 s, 65 °C for 30 s and 72 °C for 30 s; 1 cycle at 72 °C for 1 min) and purified with AMPureXP beads (Beckman Coulter, Pasadena, CA). The purified libraries were quantified using a KAPA library quantification kit. The libraries were loaded on cBot (Illumina, San Diego, CA) at a final concentration of 10 pM to perform cluster generation, followed by 2 × 76 bp sequencing on HiSeq 2000 (Illumina).

Sequencing reads were mapped to reference human genome sequence (NCBI 36.1 [hg19] assembly by TopHat (Version 2.0.6). The number of fragments in each known gene from RefSeq database (downloaded from UCSC Genome Browser on March 9, 2012) was enumerated using htseq-count from HTSeq package (version 0.5.4p9). Genes with less than ten fragments in all the samples were removed before differential expression analysis. The differential expression between conditions was statistically assessed by R/Bioconductor package edgeR (version 3.0.8). Genes with FDR of ≤0.05 and >200 bp were called as differentially expressed.

For Gene Ontology analysis, IPA (Qiagen, Valencia, CA) and GSEA (Broad Institute) were performed. We followed the standard procedure (http://www.broadinstitute.org/gsea/doc/GSEAUserGuideFrame.html) as described by GSEA user guide and used curated gene set C2 of the Molecular Signature Database version 4.0 to compute overlaps between our gene set and gene sets in Molecular Signature Database. Some of the data sets presented in the text are annotated here. For example, Wong *et al*.^64^ identified a core ESC-like gene module containing genes coordinately upregulated in mouse ESC that are shared with the human ESC-like module (Fig. 4d). In another study, Lim *et al*.^65^ identified a gene signature (Fig. 4e) that was consistently downregulated in mammary stem cells in both mouse and human. Through whole-genome DNA microarrays in 31 breast cancer cell lines, Charafer-Jauffret *et al*.^66^ found a list of genes (Fig. 4i) that was upregulated in luminal-like breast cancer cell lines compared with the mesenchymal-like breast cancer lines. Onder *et al*.^67^ performed gene expression analysis in HMLE cells (immortalized non-transformed mammary epithelium) after E-cadherin (CDH1) knockdown by RNA interference and identified genes that were downregulated after loss of E-cadherin (Fig. 4j). To analyse EMT-causing pathways in tumorigenesis, Aigner *et al*.^68^ identified a gene signature that was upregulated in MDA-MB-231 breast cancer cells after knockdown of ZEB1 by RNA interference (Fig.4k) representing transcriptional targets of E-cadherin repressor ZEB1 in invasive human cancer cells. The miR-141 downregulated genes in PCa cells were found to be greatly enriched in EZH2-repressed genes reported by Lu *et al*.^69^ and Nuytten *et al*.^70^ (Supplementary Fig. 6f). Finally, the predicted miR-141 targets in PCa cells significantly overlapped with a cohort of SUZ12 targets in embryonic stem cells^71^ (Supplementary Fig. 8a).

### Merging our RNA-Seq data with the miR-200a Ago-HITS-CLIP-Seq data

miR-200 Ago-HITS-CLIP results^12^ were available at https://bitbucket.org/sacgf/bracken_hits-clip_2013. We used the authors' in-house scripts (also available through the above link) for sequence alignment and peak calling. The miR-200a CLIP-Seq results containing the binding information were further extracted and merged with our DU145 or LAPC9 RNA-Seq data by gene symbol. Genes that were shared on both lists were called out to generate Supplementary Data 2 and 4.

### siRNA-mediated knockdown

siRNAs targeting RAC1, CDC42 and EZH2 were purchased from Origene (Rockville, MD). Three unique 27mer siRNA duplexes for each target were provided. siRNAs were transfected at 10 nM for 48 h using Lipofectamine RNAiMax (Life Technologies). Knockdown efficiency was determined by qPCR. The sequences for all siRNAs are listed in Supplementary Table 3.

### Statistical analyses

In general, experiments were done in triplicates for each condition when feasible. Results are presented as mean±s.d. calculated using Microsoft Excel. Statistical differences were determined using unpaired two-tailed Student's *t*-test for most analyses, except for tumour incidence for which we used *χ*^2^ test. *P*-values <0.05 are considered statistically significant. No statistical method was used to pre-determine sample size and no samples were excluded for any analysis.

### Data availability

The RNA-Seq data discussed in this publication have been deposited in NCBI Gene Expression Omnibus with the accession number GSE71756. All other relevant data are available from the corresponding authors upon request.

## Additional information

**How to cite this article:** Liu, C. *et al*. MicroRNA-141 suppresses prostate cancer stem cells and metastasis by targeting a cohort of pro-metastasis genes. *Nat. Commun.* **8,** 14270 doi: 10.1038/ncomms14270 (2017).

**Publisher's note**: Springer Nature remains neutral with regard to jurisdictional claims in published maps and institutional affiliations.

## Supplementary Material

Supplementary Information Supplementary Figures, Supplementary Tables and Supplementary Note.

Supplementary Data 1 Genes downregulated (Tab 1) and upregulated (Tab 2) by FDR<0.05 in Du145 cells over-expressing miR-141 in RNA-Seq analysis.

Supplementary Data 2 Putative direct targets of miR-141 revealed by merging Du145 RNA-seq data with miR-200a Ago-HITS-CLIP data.

Supplementary Data 3 Genes Downregulated (Tab 1) and upregulated (Tab 2) by FDR<0.05 in LAPC9 cells over-expressing miR-141 in RNA-Seq analysis.

Supplementary Data 4 Putative direct targets of miR-141 revealed by merging LAPC9 RNA-seq data with miR-200a Ago-HITS-CLIP data.
