# Supplementary material for: MicroRNA-141 suppresses prostate cancer stem cells and metastasis by targeting a cohort of pro-metastasis genes
Source: Nat Commun. 2017 Jan 23;8:14270. doi: 10.1038/ncomms14270 (PMC5264244; doi:10.1038/ncomms14270)
Supplement: Supplementary Information — Supplementary Figures, Supplementary Tables and Supplementary Note. [file ncomms14270-s1.pdf]

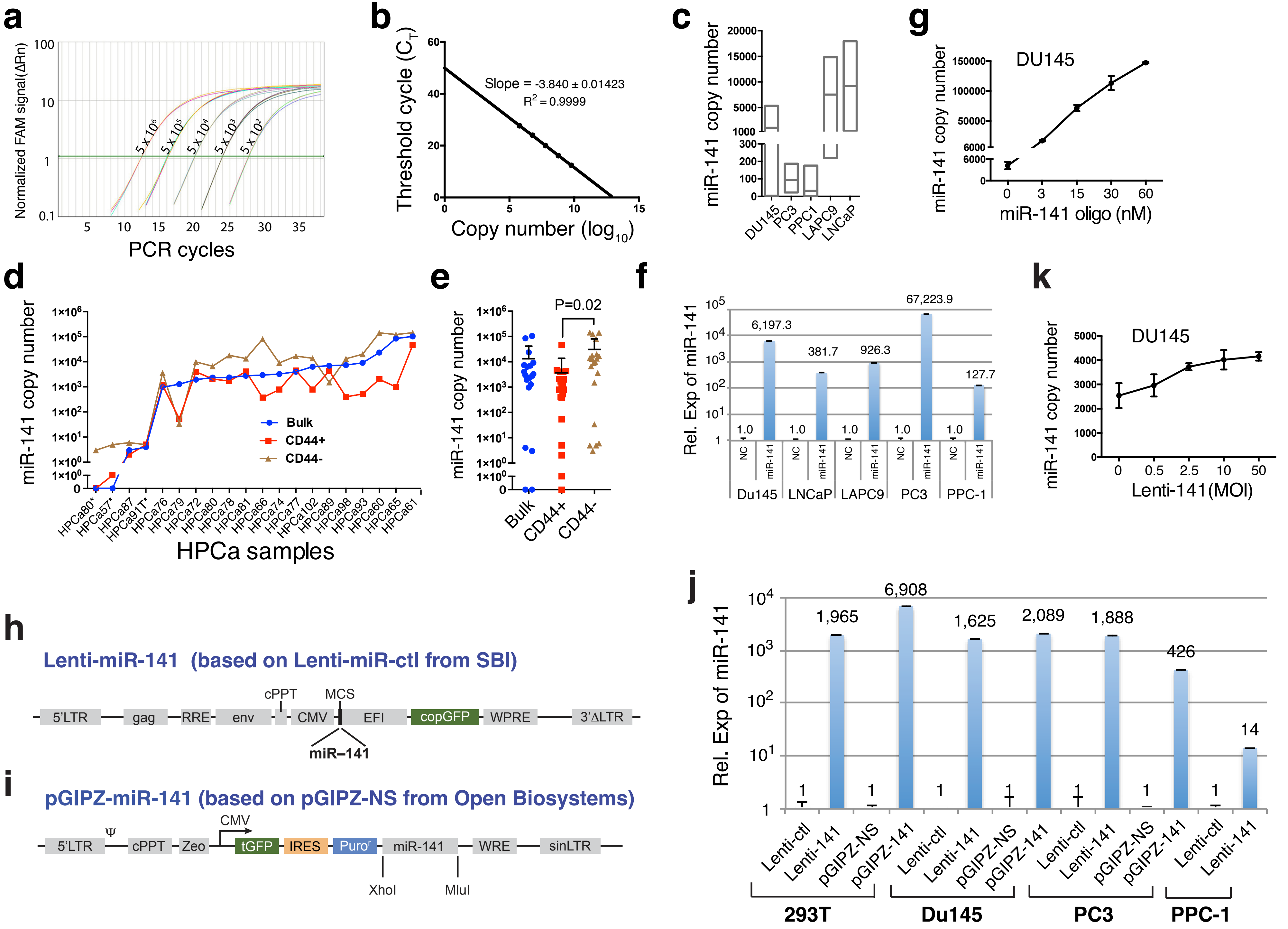

a
Normalized FAM signal(ΔRn)
PCR cycles
5x10^6
5x10^5
5x10^4
5x10^3
5x10^2
b
Threshold cycle (C_T)
Copy number (log10)
Slope = -3.840 ± 0.01423
R² = 0.9999
c
miR-141 copy number
DU145
PC3
PPC1
LAPC9
LNCaP
g
DU145
miR-141 copy number
miR-141 oligo (nM)
d
miR-141 copy number
Bulk
CD44+
CD44-
HPCa samples
e
miR-141 copy number
P=0.02
Bulk
CD44+
CD44-
f
Rel. Exp of miR-141
6,197.3
381.7
926.3
67,223.9
127.7
NC
miR-141
Du145
LNCaP
LAPC9
PC3
PPC-1
k
DU145
miR-141 copy number
Lenti-141 (MOI)
h
Lenti-miR-141 (based on Lenti-miR-ctl from SBI)
5'LTR
gag
RRE
env
cPPT
CMV
MCS
EFI
copGFP
WPRE
3'ΔLTR
miR-141
i
pGIPZ-miR-141 (based on pGIPZ-NS from Open Biosystems)
5'LTR
Ψ
cPPT
Zeo
CMV
tGFP
IRES
Puro^r
miR-141
WRE
sinLTR
XhoI
MluI
j
Rel. Exp of miR-141
1,965
6,908
1,625
2,089
1,888
426
14
Lenti-ctl
Lenti-141
pGIPZ-NS
pGIPZ-141
293T
Du145
PC3
PPC-1

Supplementary Figure 1. *Quantification of endogenous and overexpressed miR-141*. Related to Fig. 1.

a-b. Amplification plots of synthetic miR-141. Copy numbers were determined by the weight and amount of synthetic miR-141 (http://www.endmemo.com/ bio/dnacopynum.php). Shown in b is the standard curve of miR-141 in which C_T values were plotted against copy numbers.

c. Estimated endogenous miR-141 copy numbers in 5 PCa cell types, i.e., DU145 (n=11 independent experiments with triplicate samples in each measurement; copy numbers ranged from 4-5351 copies/cell), PC3 (n=3; 22-187 copies/cell), PPC-1 (n=6; 21-76 copies/cell), LNCaP (n=4; 220-14,837 copies/cell), and LAPC9 (n=2; 448-17,940 copies/cell).

d-e. Estimated miR-141 copy numbers in 20 HPCa patient tumor-derived bulk and $CD44^+$/$CD44^-$ cells (also see Fig. 1c). Shown in e is the summary of the miR-141 copy numbers (bulk, 0-103,800 copies/cell; $CD44^+$, 0-46,015; $CD44^-$, 3-145,074 copies/cell).

f. qPCR quantification of miR-141 in PCa cells transfected with miR-141 or NC oligos. RNU48 was included as the internal control. Bars represent the mean ± SD (n=3 independent experiments)

g. Dose –dependent increase in miR-141 copy numbers in DU145 cells transfected with increased concentration of transfected oligos. Copy numbers were estimated from the standard curve (see Supplementary Fig. 1a). Bars represent the mean ± SD (n=3 independent experiments)

h. Schematic of the lentiviral vector (i.e., Lenti-141) to over-express miR-141. A 316 bp sequence harboring the miR-141 stem loop (95 bp) together with flanking regions (upstream 96 bp and downstream 125bp) is cloned into the XbaI and NotI sites of the vector (System Biosciences). The empty vector was used as control (i.e., Lenti-ctl).

i. Schematic of lentiviral vector (i.e., pGIPZ-141) for over-expressing miR-141. The same 316 bp sequence harboring the miR-141 stem loop is cloned into the XhoI and MluI sites of the pGIPZ lentiviral vector (Open Biosystems). The same vector with a non-silencing 'shRNA-Mir' sequence (i.e., pGIPZ-NS) was the control vector.

j. qPCR quantification of miR-141 in PCa cells (293T cells used as a control cell type) infected with control vectors or miR-141 encoding lentiviruses in cells harvested at 72 h post infection. The relative miR-141 expression levels are indicated. Bars represent the mean ± SD (n=3).

k. Copy numbers of miR-141 in DU145 cells infected with the Lenti-141 viruses at the indicated MOI. Bars represent the mean ± SD (n=3).

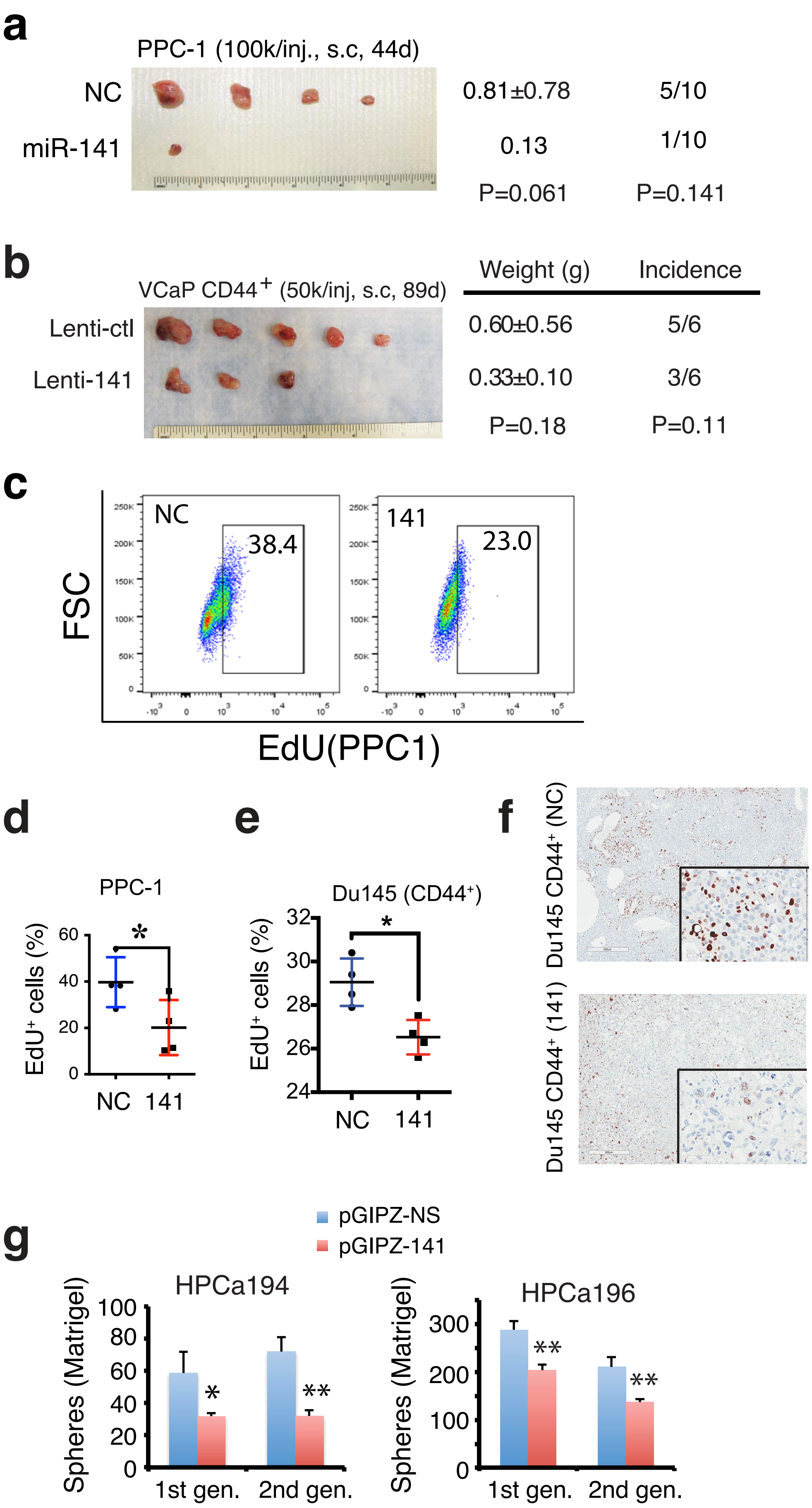
a
PPC-1 (100k/inj., s.c, 44d)
NC
miR-141
0.81±0.78
5/10
0.13
1/10
P=0.061
P=0.141
b
VCaP CD44+ (50k/inj, s.c, 89d)
Weight (g)
Incidence
Lenti-ctl
Lenti-141
0.60±0.56
5/6
0.33±0.10
3/6
P=0.18
P=0.11
c
NC
38.4
141
23.0
FSC
EdU(PPC1)
d
PPC-1
EdU+ cells (%)
NC
141
e
Du145 (CD44+)
EdU+ cells (%)
NC
141
f
Du145 CD44+ (NC)
Du145 CD44+ (141)
g
pGIPZ-NS
pGIPZ-141
HPCa194
Spheres (Matrigel)
1st gen.
2nd gen.
HPCa196
Spheres (Matrigel)
1st gen.
2nd gen.

Supplementary Figure 2. *Tumor- and proliferation-inhibitory effects of miR-141*. Related to Fig. 1.

a. miR-141 inhibits PPC-1 tumor development. Mean weight (mean ± SD), tumor incidence as well as the P values for mean weight and incidence are indicated.

b. miR-141 inhibits VCaP tumor regeneration from purified $CD44^+$ cells. Mean tumor weight (mean ± SD), tumor incidence (tumor outgrowth/total injection number) as well as the P values are indicated.

c-e. miR-141 inhibits proliferation of PCa cells measured by EdU incorporation assays. c&d, EdU incorporation assays in bulk PPC-1 cells. Shown are a representative flow histogram (c) and quantification of EdU^+ cells (d; bars represent the mean ± SD; n=5). i, miR-141 inhibits EdU incorporation in $CD44^+$ DU145 cells (bars represent the mean ± SD; n=3).

f. Ki-67 IHC staining in representative endpoint tumors derived from the DU145 $CD44^+$ cells transfected with NC (top) or miR-141 (141; bottom) oligos, respectively (also see Fig. 1g). Similar differences were observed in DU145 Lenti-141/Lenti-ctl (Fig. 1i) and in PPC-1 Lenti-141/Lenti-ctl (Fig. 1j) tumors.

g. Sphere formation assays in two primary patient tumor-derived cells (i.e., HPCa194 and HPCa196) over-expressing miR-141. Values represents mean± SD (n=3 independent experiments). *P<0.05, **P<0.01. For the second generation, spheres from the first generation were harvested and digested with Collagenase and Trypsin into single cells and re-infected with pGIPZ-NS/pGIPZ-141 lentivirus (MOI 10; 48 h).

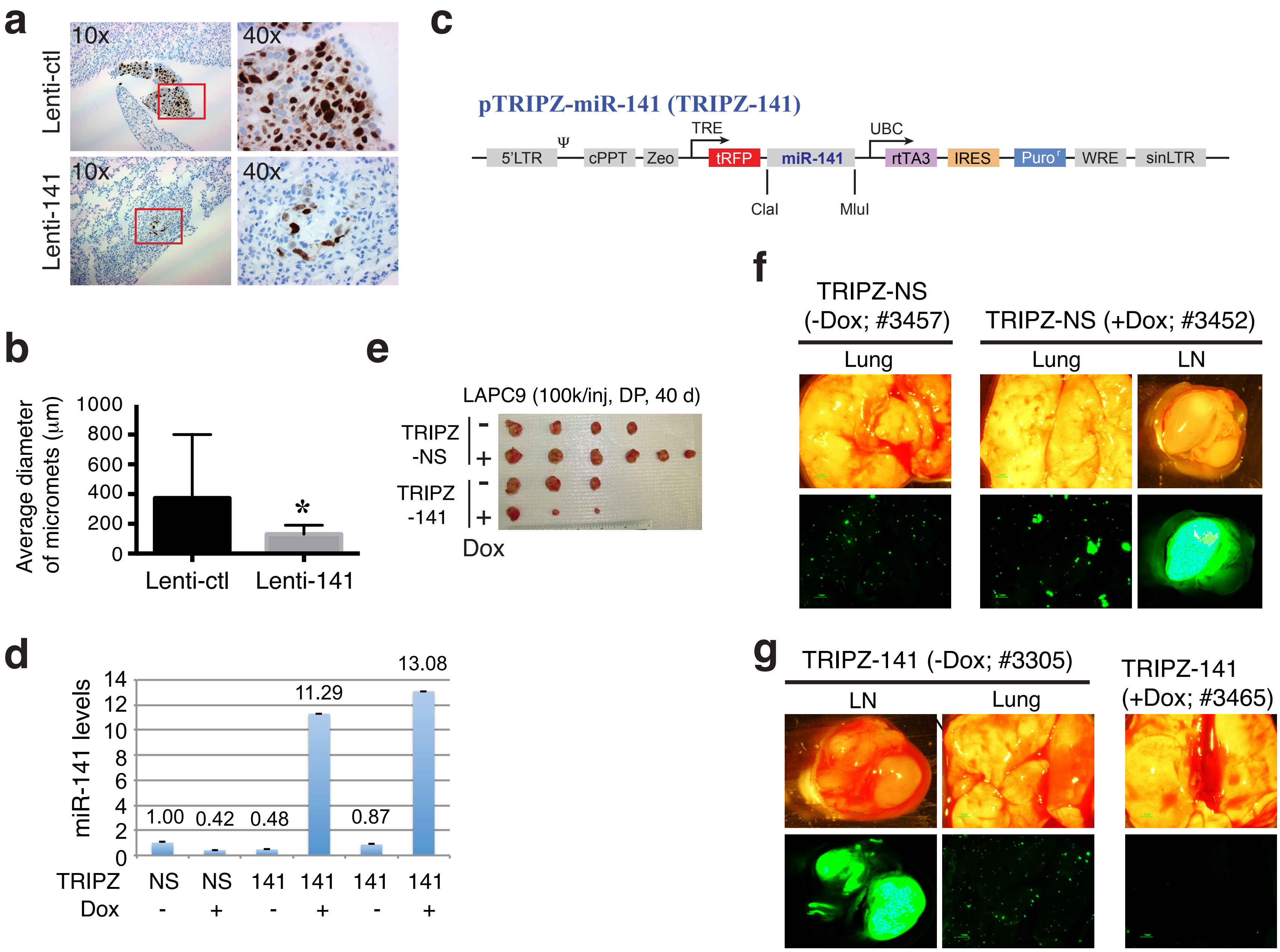

a
10x
40x
Lenti-ctl
Lenti-141
b
Average diameter of micromets (µm)
1000
800
600
400
200
0
*
Lenti-ctl
Lenti-141
c
pTRIPZ-miR-141 (TRIPZ-141)
5'LTR
Ψ
cPPT
Zeo
TRE
tRFP
miR-141
ClaI
MluI
UBC
rtTA3
IRES
Puro^r
WRE
sinLTR
d
miR-141 levels
14
12
10
8
6
4
2
0
1.00
0.42
0.48
11.29
0.87
13.08
TRIPZ
NS
NS
141
141
141
141
Dox
-
+
-
+
-
+
e
LAPC9 (100k/inj, DP, 40 d)
TRIPZ -NS
TRIPZ -141
-
+
-
+
Dox
f
TRIPZ-NS (-Dox; #3457)
TRIPZ-NS (+Dox; #3452)
Lung
Lung
LN
g
TRIPZ-141 (-Dox; #3305)
TRIPZ-141 (+Dox; #3465)
LN
Lung

Supplementary Figure 3. *miR-141 inhibits PCa metastasis*. Related to Fig. 2.

a-b. miR-141 inhibits the lung metastasis of $CD44^+$ DU145 cells. Shown in b are representative IHC images of human-specific Ki-67 staining. Shown in the bar graph (b) is the quantification of average diameter of micro-metastases in the lung for each group (n=3-5; *P<0.05).

c. Schematic of the Dox-inducible pTRIPZ-miR-141 lentiviral expression system (TRIPZ-141). The same 316 bp sequence as in Lenti-141 was cloned into the ClaI and MluI sites of the pTRIPZ inducible lentiviral vector (Open Biosystems).

d. qPCR quantification of miR-141 in DU145 cells infected with inducible TRIPZ-141 or TRIPZ-NS (non-silencing control) vector (MOI 5) with or without doxycycline (Dox; 2 μg/ml, 72 h). Relative miR-141 expression levels compared with TRIPZ-NS without Dox induction were indicated above each bar. Values represents mean± SD (n=3 independent experiments).

e. Dox-inducible expression of miR-141 suppresses LAPC9 tumor growth. 16 days post tumor cell implantation, Dox treatment (200 mg/kg body weight in feed) was initiated. Tumor incidence, mean weight and P values are presented as a bar graph in Fig. 2g.

f. Representative lung and lymph node (LN) images (top, phase; bottom, GFP) in TRIPZ-NS animals with or without Dox. Animal numbers (#) are indicated in the parentheses. Note that the numbers of GFP^+ metastatic foci in the TRIPZ-NS/-Dox vs. TRIPZ-NS/+Dox lungs were not significantly different although in the example shown the TRIPZ-NS/+Dox lung the GFP^+ foci appeared to cluster together to form larger foci.

g. Representative lung and LN images (top, phase; bottom, GFP) in TRIPZ-141 animals with or without Dox. Animal numbers are indicated in the parentheses. Note that the TRIPZ-141/+Dox lung showed much less GFP^+ foci than the TRIPZ-141/-Dox lung (also see Fig. 2i for quantification).

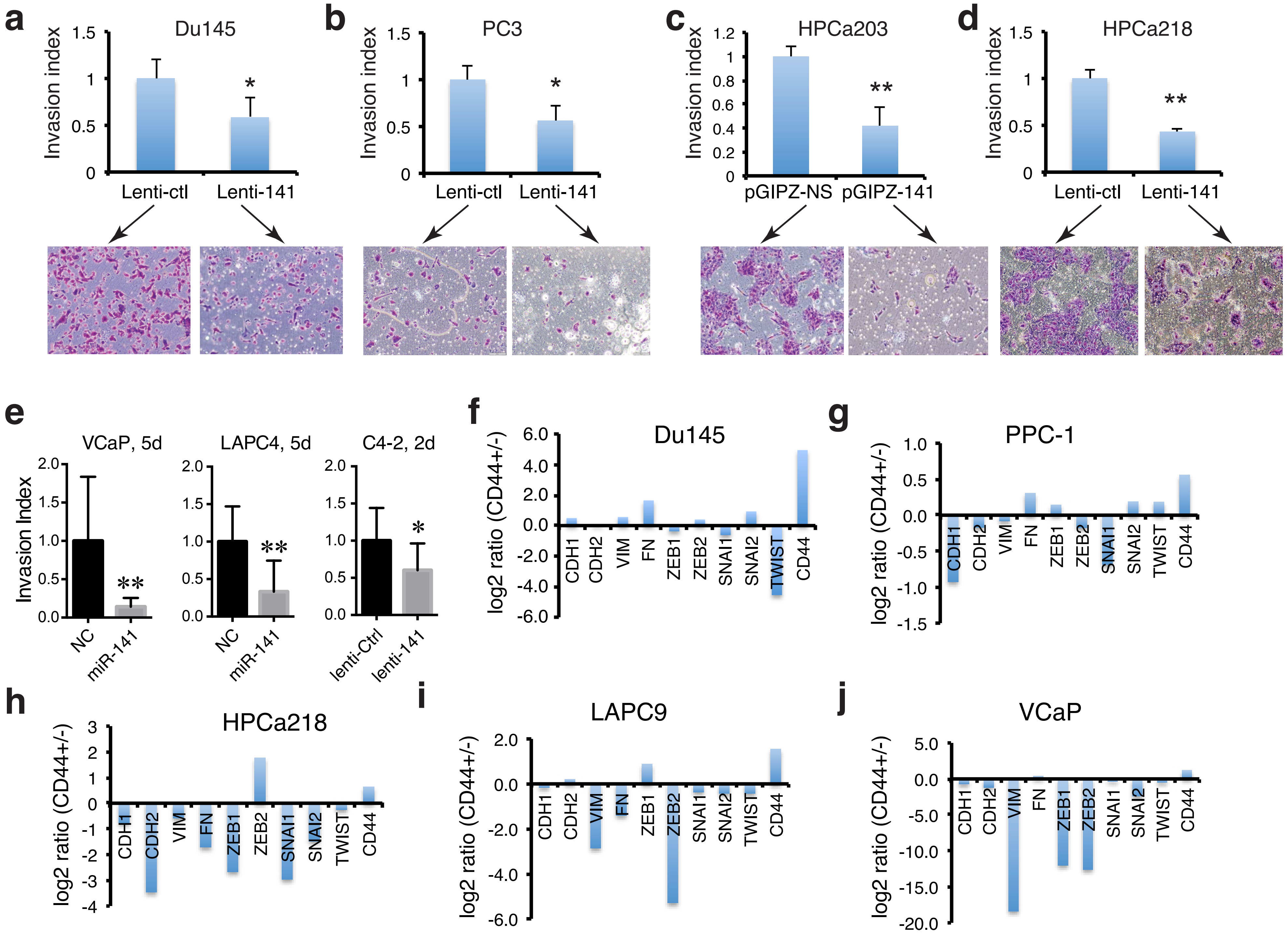
a
Du145
Invasion index
1.5
1
0.5
0
*
Lenti-ctl
Lenti-141
b
PC3
Invasion index
1.5
1
0.5
0
*
Lenti-ctl
Lenti-141
c
HPCa203
Invasion index
1.2
1
0.8
0.6
0.4
0.2
0
**
pGIPZ-NS
pGIPZ-141
d
HPCa218
Invasion index
1.5
1
0.5
0
**
Lenti-ctl
Lenti-141
e
VCaP, 5d
LAPC4, 5d
C4-2, 2d
Invasion Index
2.0
1.5
1.0
0.5
0.0
**
**
*
NC
miR-141
NC
miR-141
lenti-Ctrl
lenti-141
f
Du145
log2 ratio (CD44+/-)
6.0
4.0
2.0
0.0
-2.0
-4.0
-6.0
CDH1
CDH2
VIM
FN
ZEB1
ZEB2
SNAI1
SNAI2
TWIST
CD44
g
PPC-1
log2 ratio (CD44+/-)
1.0
0.5
0.0
-0.5
-1.0
-1.5
CDH1
CDH2
VIM
FN
ZEB1
ZEB2
SNAI1
SNAI2
TWIST
CD44
h
HPCa218
log2 ratio (CD44+/-)
3
2
1
0
-1
-2
-3
-4
CDH1
CDH2
VIM
FN
ZEB1
ZEB2
SNAI1
SNAI2
TWIST
CD44
i
LAPC9
log2 ratio (CD44+/-)
2.0
0.0
-2.0
-4.0
-6.0
CDH1
CDH2
VIM
FN
ZEB1
ZEB2
SNAI1
SNAI2
TWIST
CD44
j
VCaP
log2 ratio (CD44+/-)
5.0
0.0
-5.0
-10.0
-15.0
-20.0
CDH1
CDH2
VIM
FN
ZEB1
ZEB2
SNAI1
SNAI2
TWIST
CD44

Supplementary Figure 4. *miR-141 inhibits PCa cell invasion*. Related to Fig. 3.

a-e. Overexpression of miR-141 suppresses invasion of AR^- PCa cells (a and b), primary patient tumor-derived HPCa cells (c and d), as well as AR^+ PCa cells (e). DU145 (a) and PC3 (b), as well as two primary patient tumor (HPCa203 and HPCa218; c and d) derived cells were infected with Lenti-141 or Lenti-ctl (MOI 10; 48 h) and then used in Matrigel invasion assays. Representative images were shown below. For 3 AR^+ PCa cell types, cells were transfected with NC or miR-141 oligos (30 nM) for 5 days (5d), 5d and 2d, respectively, followed by scoring invaded cells 48 h later. Bars, mean±SEM (n=3 independent experiments). *P<0.05; **P<0.01.

f-j. qPCR analysis of 9 EMT marker genes in $CD44^+$/$CD44^-$ cells purified from 2 PCa cell cultures (e & f), 2 xenografts (g & h) and 1 primary patient tumor sample (i). The expression levels of individual genes were presented as the log2 ratio in $CD44^+$ cells over the corresponding $CD44^-$ cells.

a

b

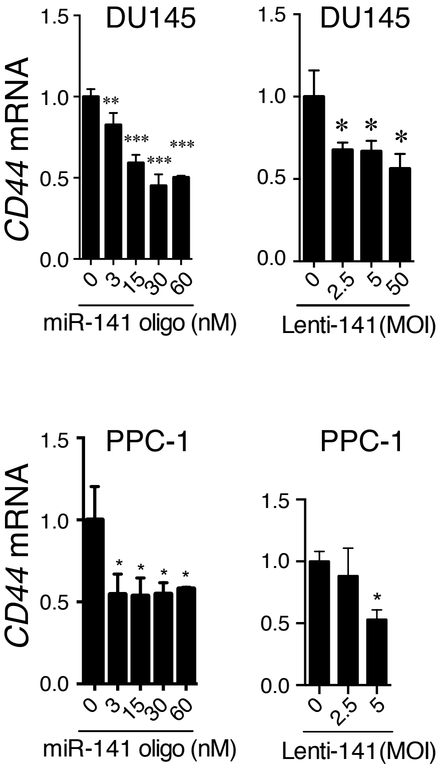
DU145
CD44 mRNA
1.5
1.0
0.5
0.0
0
3
15
30
60
miR-141 oligo (nM)
DU145
0
2.5
5
50
Lenti-141(MOI)
PPC-1
miR-141 oligo (nM)
PPC-1
Lenti-141(MOI)

Supplementary Figure 5. *miR-141 inhibits CD44 mRNA*. Related to Fig. 3.

CD44 mRNA levels were reduced in DU145 (a) and PPC-1 (b) cells transfected with miR-141 mimics (left) or infected with Lenti-141 vectors (right). The oligo concentrations and the infection MOI were indicated below. Shown were the relative miR-141 levels 72 h after transfection or infection compared to untransfected or uninfected cells (n=3-4 independent experiments). *P<0.05; **P<0.01; ***P<0.001.

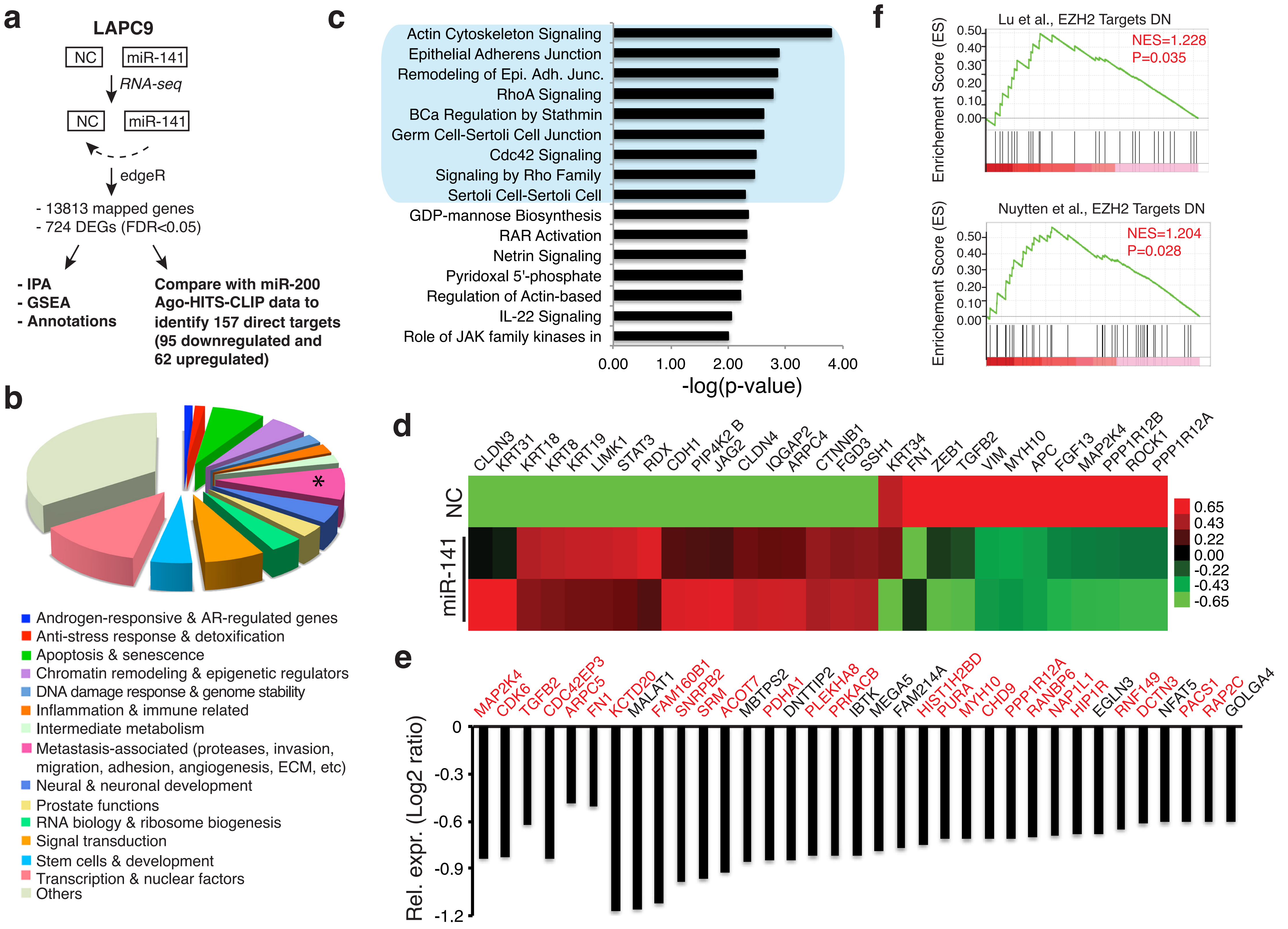
a
LAPC9
NC
miR-141
RNA-seq
NC
miR-141
edgeR
- 13813 mapped genes
- 724 DEGs (FDR<0.05)
- IPA
- GSEA
- Annotations
Compare with miR-200 Ago-HITS-CLIP data to identify 157 direct targets (95 downregulated and 62 upregulated)
b
*
Androgen-responsive & AR-regulated genes
Anti-stress response & detoxification
Apoptosis & senescence
Chromatin remodeling & epigenetic regulators
DNA damage response & genome stability
Inflammation & immune related
Intermediate metabolism
Metastasis-associated (proteases, invasion, migration, adhesion, angiogenesis, ECM, etc)
Neural & neuronal development
Prostate functions
RNA biology & ribosome biogenesis
Signal transduction
Stem cells & development
Transcription & nuclear factors
Others
c
Actin Cytoskeleton Signaling
Epithelial Adherens Junction
Remodeling of Epi. Adh. Junc.
RhoA Signaling
BCa Regulation by Stathmin
Germ Cell-Sertoli Cell Junction
Cdc42 Signaling
Signaling by Rho Family
Sertoli Cell-Sertoli Cell
GDP-mannose Biosynthesis
RAR Activation
Netrin Signaling
Pyridoxal 5'-phosphate
Regulation of Actin-based
IL-22 Signaling
Role of JAK family kinases in
0.00 1.00 2.00 3.00 4.00
-log(p-value)
d
CLDN3 KRT31 KRT18 KRT8 KRT19 LIMK1 STAT3 RDX CDH1 PIP4K2B JAG2 CLDN4 IQGAP2 ARPC4 CTNNB1 FGD3 SSH1 KRT34 FN1 ZEB1 TGFB2 VIM MYH10 APC FGF13 MAP2K4 PPP1R12B ROCK1 PPP1R12A
NC
miR-141
0.65
0.43
0.22
0.00
-0.22
-0.43
-0.65
e
MAP2K4 CDK6 TGFB2 CDC42EP3 ARPC5 FN1 KCTD20 MALAT1 FAM160B1 SNRPB2 SRM ACOT7 MBTPS2 PDHA1 DNTTIP2 PLEKHA8 PRKACB IBTK MEGA5 FAM214A HIST1H2BD PURA MYH10 CHD9 PPP1R12A RANBP6 NAP1L1 HIP1R EGLN3 RNF149 DCTN3 NFAT5 PACS1 RAP2C GOLGA4
Rel. expr. (Log2 ratio)
0
-0.3
-0.6
-0.9
-1.2
f
Lu et al., EZH2 Targets DN
NES=1.228
P=0.035
Enrichement Score (ES)
0.50
0.40
0.30
0.20
0.10
0.00
Nuytten et al., EZH2 Targets DN
NES=1.204
P=0.028
Enrichement Score (ES)
0.50
0.40
0.30
0.20
0.10
0.00

Supplementary Figure 6. *miR-141 enforces epithelial phenotype and targets a cohort of migration/invasion/metastasis related genes in LAPC9 cells*. Related to Fig. 4.

a. Experimental scheme of RNA-Seq in LAPC9 cells and subsequent data analysis.

b. Functional classification of the 459 genes downregulated by miR-141 in LAPC9 cells. The non-redundant functional classification was conducted as in Fig. 4h. Note that the gene category classified as the "Metastasis-associated" represents one of the largest affected by miR-141.

c. IPA revealed that miR-141 impacted pathways involving Rho GTPases, and actin cytoskeleton, cell-cell junction, and EMT signaling.

d. Heat map of representative EMT-related molecules in the LAPC9 RNA-Seq experiment.

e. The top 35 putative direct targets of miR-141 as revealed by merging LAPC9 RNA-Seq data with the miR-200 Ago-HITS-CLIP data. Bars represent the relative expression levels of each gene from RNA-Seq. Targets that are shared with the putative direct targets in DU145 cells are colored in red.

f. Two "EZH2 Targets Downregulated" (DN) gene sets positively correlated with the miR-141 expressing LAPC9 gene expression profile.

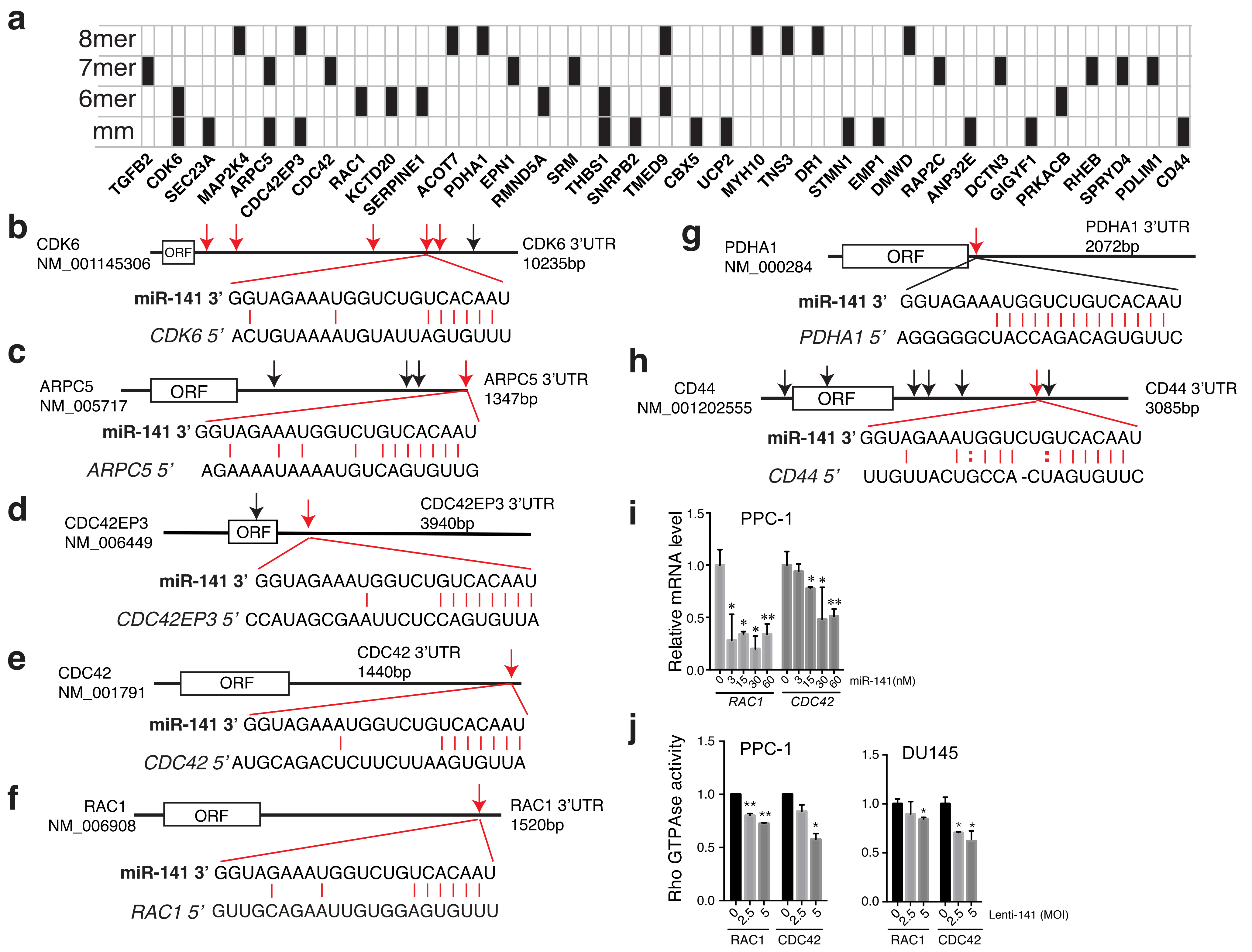
a
8mer
7mer
6mer
mm
TGFB2 CDK6 SEC23A MAP2K4 ARPC5 CDC42EP3 CDC42 RAC1 KCTD20 SERPINE1 ACOT7 PDHA1 EPN1 RMND5A SRM THBS1 SNRPB2 TMED9 CBX5 UCP2 MYH10 TNS3 DR1 STMN1 EMP1 DMWD RAP2C ANP32E DCTN3 GIGYF1 PRKACB RHEB SPRYD4 PDLIM1 CD44
b
CDK6
NM_001145306
ORF
CDK6 3'UTR
10235bp
miR-141 3' GGUAGAAAUGGUCUGUCACAAU
CDK6 5' ACUGUAAAAUGUAUUAGUGUUU
c
ARPC5
NM_005717
ORF
ARPC5 3'UTR
1347bp
miR-141 3' GGUAGAAAUGGUCUGUCACAAU
ARPC5 5' AGAAAAUAAAAUGUCAGUGUUG
d
CDC42EP3
NM_006449
ORF
CDC42EP3 3'UTR
3940bp
miR-141 3' GGUAGAAAUGGUCUGUCACAAU
CDC42EP3 5' CCAUAGCGAAUUCUCCAGUGUUA
e
CDC42
NM_001791
ORF
CDC42 3'UTR
1440bp
miR-141 3' GGUAGAAAUGGUCUGUCACAAU
CDC42 5' AUGCAGACUCUUCUUAAGUGUUA
f
RAC1
NM_006908
ORF
RAC1 3'UTR
1520bp
miR-141 3' GGUAGAAAUGGUCUGUCACAAU
RAC1 5' GUUGCAGAAUUGUGGAGUGUUU
g
PDHA1
NM_000284
ORF
PDHA1 3'UTR
2072bp
miR-141 3' GGUAGAAAUGGUCUGUCACAAU
PDHA1 5' AGGGGGCUACCAGACAGUGUUC
h
CD44
NM_001202555
ORF
CD44 3'UTR
3085bp
miR-141 3' GGUAGAAAUGGUCUGUCACAAU
CD44 5' UUGUUACUGCCA -CUAGUGUUC
i
PPC-1
Relative mRNA level
1.5
1.0
0.5
0.0
0 3 15 30 60 0 3 15 30 60 miR-141(nM)
RAC1
CDC42
j
PPC-1
DU145
Rho GTPAse activity
1.5
1.0
0.5
0.0
0 2.5 5 0 2.5 5 Lenti-141 (MOI)
RAC1
CDC42

Supplementary Figure 7. *miR-141 is predicted to directly target many molecules related to cell motility, invasion, and metastasis in PCa cells*. Related to Fig. 4 & Fig. 5.

a. By merging our RNA-Seq data in DU145 cells with the miR-200 Ago-HITS-CLIP results (https://bitbucket.org/sacgf/bracken_hits-clip_2013) (see Methods), we identified 532 transcripts (263 down- and 269 up-regulated) that have binding sites to miR-200a/miR-141 (Supplementary Data 2). Presented here is the histogram of the top 35 putative direct target molecules of miR-141 (also see Fig. 4l) with the putative miR-141 binding sites indicated by vertical filled solid bars. 8mer, 7mer, and 6mer represent the 8-bp, 7-bp, and 6-bp binding sites, respectively, in the target genes that perfectly match the miR-141 seed sequence whereas 'mm' refers to the target binding site(s) (8mer, 7mer, or 6mer) that have a mismatch to the miR-141 seed sequence.

b-h. Shown are several examples of novel direct miR-141 targets identified, including cell-cycle related (CDK6, b), Rho GTPase related (ARPC5, CDC42EP3, CDC42, and RAC1; c-f), metabolism related (PDHA1; g), and CD44 (h). The black arrows indicate putative binding site(s) with a mismatch while the red arrows the perfectly matched target sites with its sequence alignment shown below. For example, the *TGFB2* mRNA has one perfectly matched 7mer binding site for miR-141 (a) whereas the *CDK6* 3'-UTR harbors 5 perfectly matched 6mer binding sites (a; b, red arrows. The sequence for one is shown below) and 1 mismatched 8mer binding site (b; black arrow). For *CD44* (a), there is one 7mer miR-141 binding site at the 3'-UTR that contains a mismatch (h, red arrow; also see Fig. 3d) and 6 other potential binding sites with a mismatch in each at the 3'-UTR or ORF (h, black arrows). For each gene, the NM number and the length of the 3'-UTR are indicated.

i-j. qPCR of *RAC1* and *CDC42* in PPC-1 cells over-expressing miR-141 with either oligo at low concentrations (i.e., 3 and 15 nM) or lenti-virus at low MOI. Bars represent the mean ± SD (n=3 independent experiments). *P<0.05; **P<0.01.

k. miR-141 inhibits Rho GTPase activities of RAC1 and CDC42 in PCa cells. PPC-1 (left) and DU145 (right) cells were infected with Lenti-141 at low MOI (i.e., 2.5 and 5) and 72 h later cells were harvested for Rho GTPase activity assays. Bars represent the mean ± SD (n=2). *P<0.05; **P<0.01.

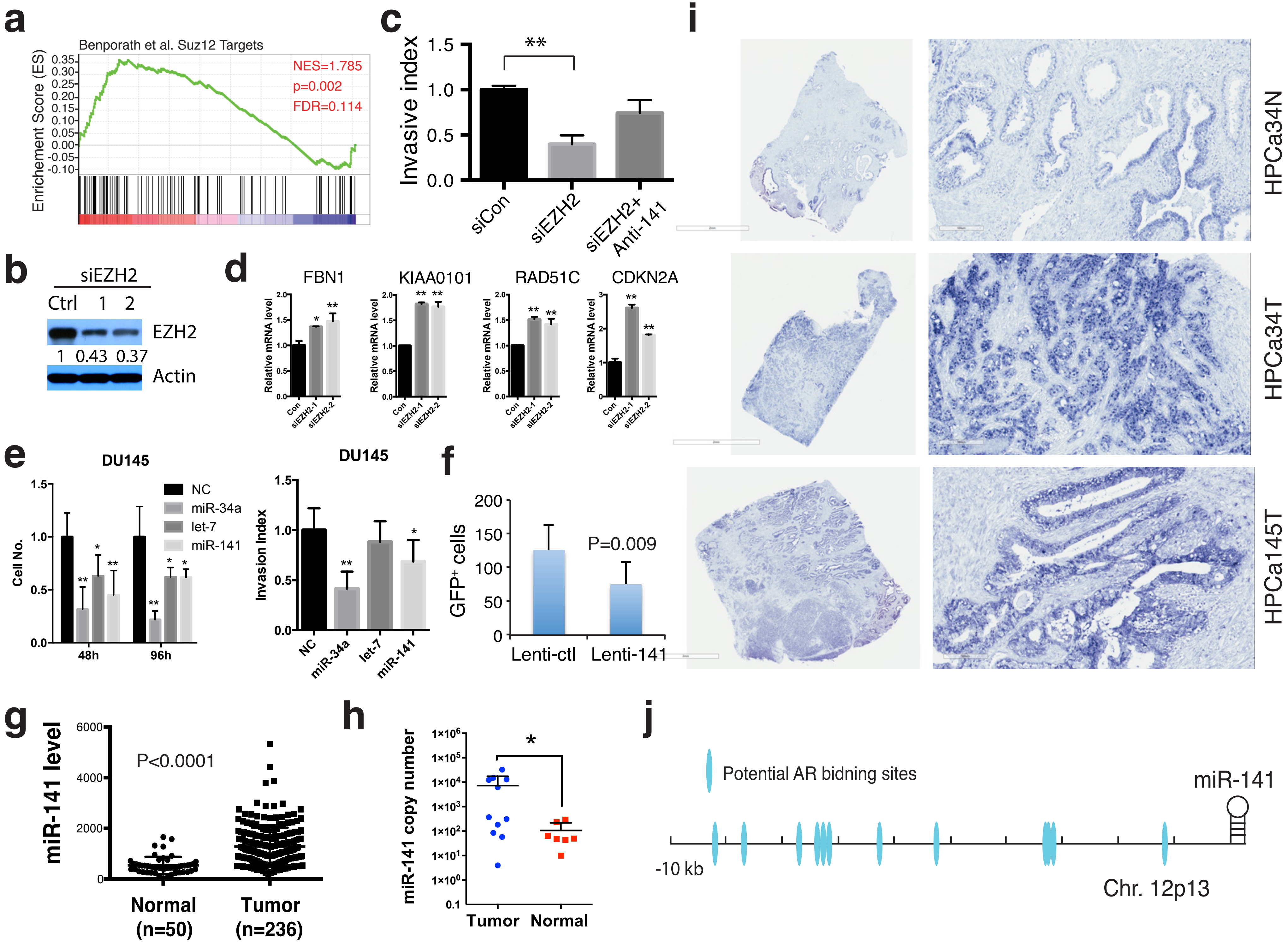
a
Benporath et al. Suz12 Targets
NES=1.785
p=0.002
FDR=0.114
Enrichement Score (ES)
b
siEZH2
Ctrl 1 2
EZH2
1 0.43 0.37
Actin
c
Invasive index
**
siCon
siEZH2
siEZH2+ Anti-141
d
FBN1
KIAA0101
RAD51C
CDKN2A
Relative mRNA level
Con
siEZH2-1
siEZH2-2
e
DU145
NC
miR-34a
let-7
miR-141
Cell No.
48h
96h
Invasion Index
f
GFP+ cells
P=0.009
Lenti-ctl
Lenti-141
g
miR-141 level
P<0.0001
Normal (n=50)
Tumor (n=236)
h
miR-141 copy number
Tumor
Normal
i
HPCa34N
HPCa34T
HPCa145T
j
Potential AR bidning sites
miR-141
-10 kb
Chr. 12p13

Supplementary Figure 8. *miR-141 is overexpressed in patient prostate tumors*. Related to Fig. 6 & Discussion.

a. GSEA showing the enrichment of miR-141 downregulated genes in the SUZ12 Targets gene set.
b. Western blotting showing the knockdown efficiency of EZH2 proteins in DU145 cells.
c. EZH2 knockdown inhibits PCa cell invasion, which can be partially overcome by anti-141. Bars, mean±SD (n=4). **P<0.01.
d. qPCR of 4 EZH2 target genes (i.e., *FBN1, KIAA0101, RAD51C*, and *CDKN2A*) in DU145 cells transfected with siEZH2-1 or siEZH2-2 (20 nM) . Bars, mean±SD (n=3). *P<0.05; **P<0.01.
e. Side-by-side comparison of the effects of miR-34a, let-7b (let-7), and miR-141 on cell viability (left) and invasion (right) in DU145 cells. Presented on the left are live cell numbers. Bars, mean±SD. *P<0.05; **P<0.01.
f. miR-141 overexpression reduces circulating tumor cells (CTCs) in tumor-bearing animals. FACS-purified $CD44^+$ DU145 cells were infected with Lenti-ctl or Lenti-141 and cells were then injected into DP of NOD/SCID mice (1.5×10^6 cells/injection). Mice were terminated on day 56 when 1 ml of whole blood was collected by cardiac puncture into K3-EDTA-coated tubes (BD Bioscience, Vacutainer) to analyze CTCs. GFP^+ DU145 cells were analyzed using FACS together with control blood from a mouse without orthotopic tumor cell injection. A total of 7 mice from Lent-ctl and 6 mice from Lenti-141 group were analyzed and presented in the bar graph are the GFP^+ cells/100,000 total cells analyzed (y-axis).
g. miR-141 levels (reads per million miRNA mapped) in the TCGA PRAD data set containing normal (n= 50) and prostate tumor (n=236) samples. P value is indicated above.
h. miR-141 copy numbers were estimated from total RNA prepared from 11 untreated HPCa samples and 7 matched normal/benign samples (tumors, 4-32,598 copies/cell; normal, 44-298 copies/cell). *P<0.05.
i. In situ hybridization (ISH) of miR-141 using pre-designed miRCURY Locked nucleotide acid (LNA) probes (Exiqon) in formalin-fixed, paraffin-embedded HPCa sections including paired benign (HPCa34N) and tumor (HPCa34T) tissues of HPCa34 (top and middle panels), as well as tumor sections from patient HPCa145 (bottom panels). Left panels, 4x; right panels, 40x.
j. Putative AR binding sites upstream (-10 kb) of the *miR-141* genomic locus on Chromosome 12p13, as predicted by the MetInspector program.

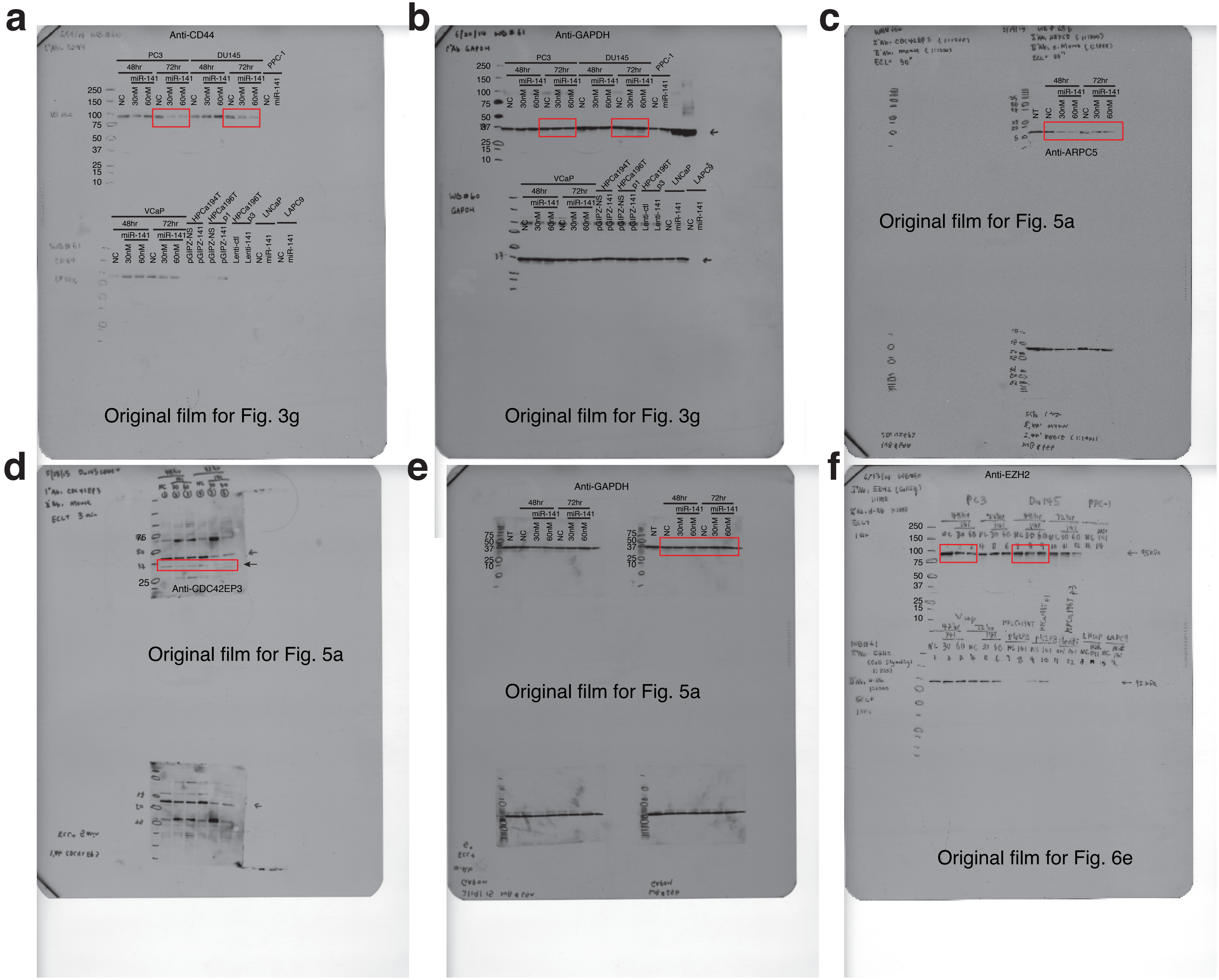

a
Anti-CD44
PC3
DU145
48hr
72hr
miR-141
PPC-1
NC
30nM
60nM
250
150
100
75
50
37
25
15
10
VCaP
HPCa194T
HPCa196T
p1
p3
LNCaP
LAPC9
pGIPZ-NS
pGIPZ-141
Lenti-ctl
Lenti-141
miR-141
Original film for Fig. 3g
b
Anti-GAPDH
Original film for Fig. 3g
c
NT
Anti-ARPC5
Original film for Fig. 5a
d
Anti-CDC42EP3
Original film for Fig. 5a
e
Anti-GAPDH
Original film for Fig. 5a
f
Anti-EZH2
Original film for Fig. 6e

Supplementary Figure 9. *Representative full films showing several targets of miR-141*.

a-b. miR-141 reduces CD44 protein levels in PC3 and DU145 cells 72 h after transfection (a). Shown in b is the GAPDH loading control. For both CD44 and GAPDH, the bands in boxed rectangles were presented in Fig. 3g.

c-e. miR-141 reduces ARPC5 (c) and CDC42EP3 (d) protein levels in DU145 cells 48 h and 72 h after transfection. Shown in e is the GAPDH loading control. For all three proteins, the bands in boxed rectangles were presented in Fig. 5a. NT, non-transfected.

f. miR-141 reduces EZH2 protein levels in PC3 and DU145 cells 48 h after transfection. The bands in boxed rectangles were presented in Fig. 6e.

Supplementary Table 1. Primers used in this study.

Taqman Primers from Life Technologies.

Primer/Probe Name	Catalog number
Has-miR-141	4427975 (ID 000463)
RNU48	4427975 (ID 001006) [control primer for Has-miR-141]
CD44	4331182 (ID Hs01075861_m1)
CDH1	4331182 (ID Hs01023894_m1)
CDH2	4331182 (ID Hs00983056_m1)
EZH2	4331182 (ID Hs00544833_m1)
Fibronectin 1	4331182 (ID Hs00365052_m1)
GAPDH	4331182 (ID Hs99999905_m1)
SNAIL1	4331182 (ID Hs00195591_m1)
SNAIL2	4331182 (ID Hs00950344_m1)
SUZ12	4331182 (ID Hs00248742_m1)
TWIST1	4331182 (ID Hs01675818_s1)
VIMENTIN	4331182 (ID Hs00185584_m1)
ZEB1	4331182 (ID Hs00232783_m1)
ZEB2	4331182 (ID Hs00207691_m1)

Other primers

Primers Name	Sequence (5' to 3')
CD44 miR-141 binding site mutagenesis primer	GTGTTGTTACTGCCACGGCGCGGCAAGTGCCTCTTGTTTTCCCAGA
CD44 miR-141 binding site mutagenesis primer Antisense	TCTGGGAAAACAAGAGGCACTTGCCGCGCCGTGGCAGTAACAACA C
EZH2 miR-141 binding site mutagenesis primer	TGTACCATACTGCATTATTGCAAAAATTCACTGGTACAATTGTGATA GCAGCTGGTGAGAAGGCAATAAAAAGTTGATTTTTAAAC
EZH2 miR-141 binding site mutagenesis primer Antisense	GTTTAAAAATCAACTTTTTATTGCCTTCTCACCAGCTGCTATCACAA TTGTACCAGTGAATTTTTGCAATAATGCAGTATGGTACA
Rac1 Forward	ACTGTCCCAACACTCCCATC
Rac1 Reverse	CAGAGGACTGCTCGGATCG
CDC42 Forward	GACAGATTACGACCGCTGAGT
CDC42 Reverse	ACTTGACAGCCTTCAGGTCA

Supplementary Table 2. Primary human PCa (HPCa) samples used in this study.

HPCa sample[a]	Age	Gleason	%CD44^{+}[b]	Purification[c]	Purity (%)[d]
HPCa57	53	7 (3+4)	50	MACS	$CD44^{+}$: 69<br>$CD44^{-}$: 95
HPCa60	54	8 (3+5)	8.7	MACS	$CD44^{+}$: ~50<br>$CD44^{-}$: N.A
HPCa61	56	6 (3+3)	5.0	MACS	$CD44^{+}$: N.A<br>$CD44^{-}$: 25
HPCa62	59	7 (4+3)	2.4	MACS	$CD44^{+}$: ~50<br>$CD44^{-}$: 100
HPCa65	59	7 (4+3)	19.9	MACS	$CD44^{+}$: 67<br>$CD44^{-}$: 100
HPCa66	58	6 (3+3)	15.0	FACS	$CD44^{+}$: NA<br>$CD44^{-}$: 94
HPCa72	58	7 (3+4)	10.2	MACS	$CD44^{+}$: 33<br>$CD44^{-}$: 100
HPCa74	59	7 (3+4)	16.2	MACS	$CD44^{+}$: 70<br>$CD44^{-}$: 100
HPCa76	64	7 (4+3)	0.02	MACS	$CD44^{+}$: ~10<br>$CD44^{-}$: 100
HPCa77	46	6 (3+3)	14.2	MACS	$CD44^{+}$: 45<br>$CD44^{-}$: 100
HPCa78	64	7 (3+4)	19.2	MACS	$CD44^{+}$: 45<br>$CD44^{-}$: 100
HPCa79	67	7 (4+3)	8.2	MACS	$CD44^{+}$: 15<br>$CD44^{-}$: 100
HPCa80	65	9 (4+5)	4.4	MACS	$CD44^{+}$: 13<br>$CD44^{-}$: 85
HPCa81	54	7 (3+4)	20.9	MACS	$CD44^{+}$: 64<br>$CD44^{-}$: 90
HPCa87*	57	9 (4+5)	N.D	MACS	$CD44^{+}$: 93<br>$CD44^{-}$: 100
HPCa89	55	9 (4+5)	24	MACS	$CD44^{+}$: 87<br>$CD44^{-}$: 90
HPCa91*	60	8 (3+5)	N.D	MACS	$CD44^{+}$: 95<br>$CD44^{-}$: 90
HPCa93	58	7 (4+3)	0.99	FACS	$CD44^{+}$: 87<br>$CD44^{-}$: 100
HPCa98	64	8 (3+5)	5.74	FACS	$CD44^{+}$: 79<br>$CD44^{-}$: 100
HPCa102	55	6 (3+3)	24.8	FACS	$CD44^{+}$: 99<br>$CD44^{-}$: 85

HPCa194	69	7 (3+4)	Used in sphere formation and clonal assays (Fig. S2a)
HPCa196	47	7 (4+3)	Used in sphere formation and clonal assays (Fig. S2a)
HPCa202	61	7 (3+4)	Used in sphere formation and clonal assays (Fig. 1k-m)
HPCa203	67	9 (4+5)	Used in invasion assays (Fig. S3c)
HPCa217	53	7 (3+4)	Used in sphere formation and invasion assays (Repeat experiments; data not shown)
HPCa218	62	7 (4+3)	Used in qPCR of EMT markers in $CD44^{+/-}$ populations, and In invasion assays (Fig. S3j)

[a]Primary HPCa samples were obtained from the robotic (Da Vinci) surgery with patient's consent. The age and Gleason score of each tumor are indicated. *indicates primary xenografts. Sample HPCa60 – HPCa102 were used in purifying out $CD44^{+}$/$CD44^{-}$ cells for validation of miR-141 expression (Fig. 1c). Samples HPCa194 – HPCa218 (below) were used in other functional assays as indicated.

[b]The % of $CD44^{+}$ HPCa cells was determined by flow analysis prior to sorting. N.D, not determined.

[c]$CD44^{+}$ and $CD44^{-}$ cells were purified out using MACS (magnetic cell sorting) or FACS (fluorescence activated cell sorting).

[d]The purity of MACS-purified cells, determined by counting $CD44^{+}$ cells under a fluorescence microscope, was variable for both $CD44^{+}$ and $CD44^{-}$ cell populations. The purity of FACS-purified cells, determined by post-sort flow analysis, was ~80-99% for $CD44^{+}$ HPCa cells and 85-100% for $CD44^{-}$ cells.

Supplementary Table 3. Antibodies and siRNAs used in this study.

Antibody	Company	Catalog number	Dilution
Actin	Sigma	A1978	1:2,500
ARPC5	Abnova	H00010092-A01	1:1,000
CD44	AbCam	Ab51037	1:500
CDC42	Cell Signaling	2466	1:1,000
CDC42EP3	Abnova	H00010602-A01	1:100
E-Cadherin	Santa Cruz	SC-7870	1:1,000
EZH2	Cell Signaling	5246	1:1,000
GAPDH	Santa Cruz	SC-25778	1:1,000
H3	Cell Signaling	9715S	1:3,000
H3K27me3	Millipore	07-449	1:500
RAC1	Cell Signaling	2465	1:1,000
SUZ12	Cell Signaling	3737	1:1,000
ZEB1	Cell Signaling	3396	1:1,000

siRNA	Company	Catalog #	Sequence
siRac1	Origene	SR303958	GGAACUAAACUUGAUCUUAGGGATG
			ACAUUGUACUGUAAUGGAGUGAGCG
			GUAGUUCUCAGAUGCGUAAAGCAGA
siCDC42	Origene	SR300714	ACAAAUUUCCAUCGGAAUAUGUACC
			CCACAAACAGAUGUAUUUCUAGUCT
			GGAGAACCAUAUACUCUUGGACUTT
siEZH2	Origene	SR301494	AGGAUACAGACAGUGAUAGGGAAGC
			GGCACUUACUAUGACAAUUUCUGTG
			GCUCUAGACAACAAACCUUGUGGAC

Supplementary Table 4. HPCa and benign samples used in Fig. S8h.

HPCa#	Gleason	Age	Tumor Stage
**HPCa157T**	7 (4+3)	53	pT3a, pN0 (50% tumor involvement)
**HPCa154T**	9 (4+5)	63	pT3b, pN0 (100% tumor involvement)
**HPCa166T**	7 (4+3)	53	pT3a, pN0 (50-60% tumor involvement)
**HPCa171T**	7 (4+3)	61	pT2c, pN0
**HPCa172T**	7 (4+3)	61	pT2c, pN0 (20% tumor involvement)
**HPCa178T**	9 (5+4)	68	pT3a, pN0 (60% tumor involvement)
**HPCa216T**	7 (3+4)	61	pT2c, pN0 (20% tumor involvement)
**HPCa218T**	7 (4+3)	62	pT3b, pN0 (15% tumor involvement)
**HPCa227T**	7	66	N/A
**HPCa228T**	7 (4+3)	71	N/A (20% tumor involvement)
**HPCa231T**	7 (4+3)	72	pT3b, pN0 (50% tumor involvement)
**HPCa163N**		62	Normal/benign tissue
**HPCa164N**		54	Normal/benign tissue
**HPCa165N**		75	Normal/benign tissue
**HPCa172N**		61	Normal/benign tissue
**HPCa174N**		69	Normal/benign tissue
**HPCa175N**		67	Normal/benign tissue
**HPCa176N**		62	Normal/benign tissue

Supplementary Note 1

Whole-genome RNA-Seq analysis in LAPC9 reveals similar signaling molecules and pathways targeted by miR-141 to those in DU145 cells

We also performed whole-genome RNA-Seq analysis in xenograft-derived LAPC9 cells (Supplementary Fig. 6) transfected with miR-141 and NC oligos (30 nM; 48 h). Of the 13,813 mapped genes, we identified 724 DEGs (FDR<0.05) including 459 down- and 265 up-regulated genes (Supplementary Fig. 6a; Supplementary Data 3). Functional annotations of the 459 downregulated genes revealed "Metastasis-associated" gene category to be a major class affected by miR-141 (Supplementary Fig. 6b). Similar to in DU145 cells, IPA demonstrated that miR-141 expression in LAPC9 cells impacted multiple pathways involved in actin cytoskeleton, adherens junction, and Cdc42/RhoA signaling (Supplementary Fig. 6c, shaded). Furthermore, miR-141 prominently upregulated many epithelial markers including E-Cadherin, Claudin members and KRTs while downregulated TGFB2, ZEB1, MAP2K4, VIM, NYH10, FN1 and many other mesenchymal genes (Supplementary Fig. 6d). GSEA positively correlated the miR-141 induced gene expression profile in LAPC9 cells with those in luminal (i.e., differentiated epithelial) breast cancer cells (data not shown).

We then merged the LAPC9 RNA-Seq data with the miR-200a CLIP-Seq data and found 157 putative direct targets of miR-141 in LAPC9 cells (Supplementary Fig. 6a; Supplementary Data 4). Remarkably, 26 of the top 35 downregulated genes, including CDK6, CDC42EP3, and PADH1 that harbor the miR-141 binding site(s) were commonly found in DU145 cells (Supplementary Fig. 6e, marked in red; Supplementary Fig. 7a-h). Finally, we found that the miR-141-induced gene expression profile in LAPC9 cells was associated with two data sets of "EZH2 Targets Downregulated" (Supplementary Fig. 6f).

Overall, miR-141 in LAPC9 cells elicits very similar gene expression changes and prominently targets genes involved in Rho GTPases, actin dynamics, EMT, and metastasis.
